# Evolution of economic burden of heart failure by ejection fraction in newly diagnosed patients in Spain

**DOI:** 10.1186/s12913-023-10376-z

**Published:** 2023-12-01

**Authors:** Carlos Escobar, Beatriz Palacios, Victoria Gonzalez, Martín Gutiérrez, Mai Duong, Hungta Chen, Nahila Justo, Javier Cid-Ruzafa, Ignacio Hernández, Phillip R. Hunt, Juan F. Delgado

**Affiliations:** 1 Cardiology Department, University Hospital La Paz, Madrid, 28046 Spain; 2 AstraZeneca Farmaceutica, Madrid, 28050 Spain; 3 Evidera, London, W6 8BJ UK; 4 AstraZeneca, Gaithersburg, MD 20878 USA; 5 Evidera, Stockholm, 113 21 Sweden; 6 Department of Neurobiology, Care Sciences, and Society, Karolinska Institute, Stockholm, 17177 Sweden; 7 Evidera, Barcelona, 08005 Spain; 8 Atrys Health, Madrid, 28002 Spain; 9 Cardiology Department, University Hospital 12 de Octubre, CIBERCV, Madrid, 28041 Spain

**Keywords:** Cost, Heart failure, Healthcare resource utilization, Hospitalization, Sacubitril/valsartan, SGLT2 inhibitors

## Abstract

**Objective:**

To describe healthcare resource utilization (HCRU) and costs, in patients with newly diagnosed heart failure (HF) according to ejection fraction (EF) in Spain.

**Methods:**

Retrospective cohort study that analyzed anonymized, integrated and computerised medical records in Spain. Patients with ≥ 1 new HF diagnosis between January 2013 and September 2019 were included and followed-up during a 4-year period. Rates per 100 person-years of HCRU and costs were estimated.

**Results:**

Nineteen thousand nine hundred sixty-one patients were included, of whom 43.5%, 26.3%, 5.1% and 25.1% had HF with reduced, preserved, mildly reduced and unknown EF, respectively. From year 1 to 4, HF rates of outpatient visits decreased from 1149.5 (95% CI 1140.8–1159.3) to 765.5 (95% CI 745.9–784.5) and hospitalizations from 61.7 (95% CI 60.9–62.7) to 15.7(14.7–16.7) per 100 person-years. The majority of HF-related healthcare resource costs per patient were due to hospitalizations (year 1–4: 63.3–38.2%), followed by indirect costs (year 1–4: 12.2–29.0%), pharmacy (year 1–4: 11.9–19.9%), and outpatient care (year 1–4: 12.6–12.9%). Mean (SD) per patient HF-related costs decreased from 2509.6 (3518.5) to 1234.6 (1534.1) Euros (50% cost reduction). At baseline, 70.1% were taking beta-blockers, 56.3% renin-angiotensin system inhibitors, 11.8% mineralocorticoid receptor antagonists and 8.9% SGLT2 inhibitors. At 12 months, these numbers were 72.3%, 65.4%, 18.9% and 9.8%, respectively.

**Conclusions:**

Although the economic burden of HF decreased over time since diagnosis, it is still substantial. This reduction could be partially related to a survival bias (sick patients died early), but also to a better HF management. Despite that, there is still much room for improvement.

**Supplementary Information:**

The online version contains supplementary material available at 10.1186/s12913-023-10376-z.

## Introduction

Heart failure (HF) is a common condition in clinical practice, with a prevalence of around 2% and an incidence of about 0.3 per 100 person-years, and increasing over time [1, 2]. Despite traditional treatments with renin angiotensin system inhibitors, beta blockers and mineralocorticoid receptor antagonists, morbidity and mortality remain high. For instance, a recent study has shown that after a 5-year follow-up period, until 2019, nearly 28% of patients with HF had died [3]. In addition, HF hospitalizations are frequent in this population and represent an inflection point in the evolution of patients with HF, as they markedly increase the risk for further complications, including death and HF rehospitalizations. Of note, in the last decade the volume of HF hospitalizations has increased over time, with a thirty-day readmission rate of around 20% [4–7].

HF is associated with a substantial economic burden, including direct health care costs (hospitalizations, outpatient care) and indirect costs, particularly, work productivity loss [8]. However, despite the large number of studies that have analyzed the socio-economic consequences of HF, further research is warranted. First, there are important differences between countries, particularly high vs low- and middle-income countries. In addition, the majority of these studies have been limited to short periods of follow-up, or have compared different years, but with a different cohort of patients each year, have not analyzed costs and healthcare resource utilization (HCRU) according to HF subtypes, nor have they considered the impact of indirect costs [8–15]. However, although the majority of guidelines recognize the high costs associated with HF, a study that includes an incident cohort of HF patients, with any ejection fraction (EF) and long follow-up seems necessary [16, 17].

The objective of the study was to describe HCRU and costs, including HF-related and all-cause, for general practitioner and specialist outpatient visits, hospitalisations, pharmacy and indirect costs, over 4 years since index date (baseline), in an overall incident HF cohort of newly diagnosed patients (de novo HF), and also stratified by EF subgroups.

## Methods

This was a retrospective cohort study that analyzed anonymized, integrated and computerised medical records from 2012 through 2019 from seven Spanish Autonomous Communities, with a total of 1.8 million patients, provided by the validated BIG-PAC database [1, 6, 11, 18]. Previous studies have proved the representativeness of the Spanish population through this database. Thus, it has been demonstrated that the age pyramids of the Spanish population and the BIG-PAC database are similar [18]. The research was conducted in accordance with the Declaration of Helsinki. The study was approved by the Research Ethics Committee of HM Hospitales, Madrid, Spain. The requirement for obtaining written informed consent was waived by the Research Ethics Committee of HM Hospitales, Madrid, Spain, as the study used secondary data fully anonymized.

Patients with ≥ 1 new inpatient or outpatient HF diagnosis (ICD-10 code) between January 2013 and September 2019 were included and followed-up during a 4-year period from diagnosis. The study included adults with at least one year of enrolment in the database prior to index date (the first qualifying HF diagnosis). The baseline period was one year prior to the index date. Patients with chronic kidney disease stage V requiring dialysis before the index date were excluded, as the management of patients on dialysis is different from the general population with HF, and also because dialysis by itself generates particularly high associated costs, which would alter the real costs of the patient with HF. HF patients were classified according to left ventricular EF at the moment of diagnosis: HF with preserved EF (HFpEF) was defined as an EF value of ≥ 50% (subtype 1: EF 50 to < 60%; subtype 2: EF ≥ 60%), HF with reduced EF (HFrEF) as an EF value of ≤ 40%, HF with mildly reduced EF (HFmrEF) as an EF value > 40% and < 50%, and HF with unspecified EF (HFuEF) included patients without echocardiograph data [7].

Biodemographic data, cardiovascular risk factors, vascular disease, and other comorbidities during the baseline period were retrieved. In addition, newly-prescribed HF treatments at baseline (at the moment of diagnosis) and among survivors after 12 months of follow-up after diagnosis were also recorded. During the study, few losses of follow-up were observed (approximately 3%). All-cause and HF-related HCRU and costs were estimated separately for each year after the index date during a 4-year period. These data included: inpatient care (number of hospitalizations > 24 h, length of hospital stays and costs estimated by the unit cost per day), outpatient care (number of visits to a general practitioner, number of visits to a specialist, costs estimated by the unit cost per general practitioner and specialist visit), pharmacy (number of prescriptions, total prescription costs estimated based on retail price + value-added tax [VAT]) and costs relating to work productivity loss (indirect cost), estimated by the unit cost per day associated to the number of work absences in days reported in primary care setting, according to the mean interprofessional wage. Outpatient costs were calculated based on standard cost for general practitioners/ specialist visits. Costs of inpatient stays were calculated based on the diagnosis-related group (DRG)-based reimbursement of the stays. Pharmacy costs were based on full price of product. Costs of absence from work were calculated by multiplying the number of days of absence from work due to sickness by the mean daily salary of a working person in Spain (available at https://www.ine.es/dynt3/inebase/index.htm?padre=4563&capsel=4563. Accessed: 15/05/2020). All costs were presented in euros. Inpatient and outpatient visits with any HF ICD-10 code were assumed to be HF-related HCRU. Health Statistics by the National Statistics Institute (INE in Spanish, www.ine.es) were used as the source for unit costs (Supplementary table 1). All data were analyzed for the overall HF population and according to EF phenotype.

### Statistical analysis

Baseline characteristics of patients were summarised using descriptive statistics. For continuous variables, the number of patients, mean and standard deviation (SD) were reported. Frequency distributions with quantity and percentages were reported for categorical variables. For years 1 to 4, the rates of HCRU with 95% confidence interval (CI), including the number of hospitalizations and outpatient visits (general practitioners and specialist overall and HF-related), were estimated by year after the index date. Person-time at risk was determined from all eligible patients with an HF diagnosis at the beginning of the calendar year. The CI for HCRU was estimated using nonparametric bootstrapping method (SciPy package) [19, 20], with the number of resampling set at 1,000. Length of inpatient stays in days, work absences in days, and number of prescriptions per patient were calculated as mean (SD), and median (IQR). All costs were estimated as mean (SD), and median (IQR) per patient. All data were analyzed using the statistical package SPSS v25.0.

## Results

A total of 19,961 patients with a new diagnosis of HF were included in the study, of whom 43.5% had HFrEF, 26.3% HFpEF, 5.1% HFmrEF and in 25.1% the EF was unknown (58.1%, 35.1% and 6.8%, respectively, when considering only those patients with known EF). In the overall HF population, mean age was 69.7 (19.0) years and 53.8% of patients were men. With regard to comorbidities, 59.1% of patients had hypertension, 27.6% type 2 diabetes, 33.1% coronary artery disease, 28.2% atrial fibrillation and 26.7% chronic kidney disease. Compared to patients with HFrEF, patients with HFpEF were older, more commonly female and had a higher prevalence of atrial fibrillation at baseline. By contrast, patients with HFrEF more frequently had type 2 diabetes, coronary artery disease, chronic kidney disease, stroke and peripheral artery disease. Patients with HFmrEF shared clinical characteristics of patients with HFrEF and HFpEF patients (Table 1).

**Table 1:** Baseline clinical characteristics in the incident HF cohort (Index Date 2013—2019)

	**HF Incident Cohort (*n* = 19,961; 100%)**	**HFrEF (*n* = 8,678; 43.5%)**	**HFmrEF (*n* = 1,022; 5.1%)**	**HFpEF (*n* = 5,244; 26.3%)**	**HFpEF (50 to < 60%) (*n* = 1,833; 9.2%)**	**HFpEF (≥ 60%) (*n* = 3,411; 17.1%)**	**HFuEF (*n* = 5,017; 25.1%)**
**Biodemographic data**							
Age. Years (SD)	69.7 (19.0)	65.6 (18.6)	72.3 (18.8)	73.4 (18.6)	73.2 (18.4)	73.5 (18.7)	72.3 (18.9)
Gender (male), n (%)	10,731 (53.8)	5,719 (65.9)	433 (42.4)	1,772 (33.8)	608 (33.2)	1,164 (34.1)	2,807 (56.0)
NYHA Functional Classification, n (%)							
Class I	2,669 (13.4)	1,158 (13.3)	129 (12.6)	670 (12.8)	238 (13.0)	432 (12.7)	712 (14.2)
Class II	8,182 (41.0)	3,040 (35.0)	456 (44.6)	2,704 (51.6)	940 (51.3)	1,764 (51.7)	1,982 (39.5)
Class III	8,274 (41.5)	4,032 (46.5)	384 (37.6)	1,749 (33.4)	619 (33.8)	1,130 (33.1)	2,109 (42.0)
Class IV	551 (2.8)	326 (3.8)	36 (3.5)	71 (1.4)	23 (1.3)	48 (1.4)	118 (2.4)
Unknown	285 (1.4)	122 (1.4)	17 (1.7)	50 (1.0)	13 (0.7)	37 (1.1)	96 (1.9)
**Cardiovascular risk factors**,** n (%)**							
Hypertension	11,793 (59.1)	5,293 (61.0)	626 (61.3)	2,947 (56.2)	1,055 (57.6)	1,892 (55.5)	2,927 (58.3)
Dyslipidemia	8,959 (44.9)	3,813 (43.9)	459 (44.9)	2,308 (44.0)	829 (45.2)	1,479 (43.4)	2,379 (47.4)
Diabetes type 1	741 (3.7)	356 (4.1)	45 (4.4)	169 (3.2)	94 (5.1)	75 (2.2)	171 (3.4)
Diabetes type 2	5,511 (27.6)	2,459 (28.3)	277 (27.1)	1,354 (25.8)	609 (33.2)	745 (21.8)	1,421 (28.3)
**Vascular disease**,** n (%)**							
Coronary artery disease	6,602 (33.1)	3,361 (38.7)	318 (31.1)	1,376 (26.2)	475 (25.9)	901 (26.4)	1,547 (30.8)
Myocardial Infarction	3,002 (15.0)	1,387 (16.0)	123 (12.0)	702 (13.4)	326 (17.8)	376 (11.0)	790 (15.8)
Atrial fibrillation	5,637 (28.2)	2,043 (23.5)	304 (29.8)	1,861 (35.5)	658 (35.9)	1,203 (35.3)	1,429 (28.5)
Chronic kidney disease	5,337 (26.7)	2,674 (30.8)	310 (30.3)	1,181 (22.5)	418 (22.8)	763 (22.4)	1,172 (23.4)
Stroke	2,014 (10.1)	1,069 (12.3)	95 (9.3)	315 (6.0)	149 (8.1)	166 (4.9)	535 (10.7)
Peripheral arterial disease	943 (4.7)	441 (5.1)	31 (3.0)	168 (3.2)	84 (4.6)	84 (2.5)	303 (6.0)

*HF* Heart failure, *HFmrEF* Heart failure with mildly reduced ejection fraction, *HFpEF* Heart Failure with preserved ejection fraction, *HFrEF* Heart Failure with reduced ejection fraction, *HFuEF* Heart Failure with unspecified ejection fractionNYHA: New York Heart Association, *SD* standard deviation

With regard to treatments, at baseline, 70.1% of patients were taking beta blockers, 56.3% renin-angiotensin system (angiotensin-converting enzyme inhibitors/angiotensin receptor II blockers: 50.2%; sacubitril-valsartan: 6.1%), 11.8% mineralocorticoid receptor antagonists and 8.9% sodium-glucose co-transporter-2 inhibitors (SGLT2i). After 12 months of follow-up, the proportion of surviving patients with these treatments increased to 72.3%, 65.4%, 18.9% and 9.8%, respectively. The prescription of disease modifying HF drugs were more frequent in patients with HFrEF than in the other HF subtypes (Table 2).

**Table 2:** Treatments^a^ (baseline and at month 12) in the incident HF cohort (Index Date 2013—2019)

	**HF Incident Cohort (*n* = 19,961; 100%)**	**HFrEF (*n* = 8,678; 43.5%)**	**HFmrEF (*n* = 1,022; 5.1%)**	**HFpEF (*n* = 5,244; 26.3%)**	**HFpEF (50 to < 60%) (*n* = 1,833; 9.2%)**	**HFpEF (≥ 60%) (*n* = 3,411; 17.1%)**	**HFuEF (*n* = 5,017; 25.1%)**
**HF drugs (baseline; *n* = 19,961)**,** n (%)**							
Diuretics	13,845 (69.4)	6,175 (71.2)	632 (61.8)	3,542 (67.5)	1,258 (68.6)	2,284 (67.0)	3,496 (69.7)
Beta-blockers	13,992 (70.1)	6,257 (72.1)	707 (69.2)	3,414 (65.1)	1,206 (65.8)	2,208 (64.7)	3,614 (72.0)
ACEi/ARB	10,026 (50.2)	4,967 (57.2)	370 (36.2)	1,856 (35.4)	636 (34.7)	1,220 (35.8)	2,833 (56.5)
Sacubitril-valsartan	1,219 (6.1)	605 (7.0)	49 (4.8)	215 (4.1)	96 (5.2)	119 (3.5)	350 (7.0)
MRA	2,360 (11.8)	1,068 (12.3)	127 (12.4)	531 (10.1)	188 (10.3)	343 (10.1)	634 (126)
SGLT2i	1,779 (8.9)	729 (8.4)	86 (8.4)	505 (9.6)	190 (10.4)	315 (9.2)	459 (9.2)
Digoxin, (%)	4,007 (20.1)	1,960 (22.6)	172 (16.8)	910 (17.4)	330 (18.0)	580 (17.0)	965 (19.2)
Ivabradine, (%)	1,218 (6.1)	616 (7.1)	37 (3.6)	249 (4.8)	106 (5.8)	143 (4.2)	316 (6.3)
Hydralazine and nitrate, (%)	14 (0.07)	7 (0.08)	1 (0.10)	4 (0.08)	3 (0.2)	1 (0.0)	2 (0.04)
**HF drugs (12 months; *n* = 19,309)**,** n (%)**							
Diuretics, (%)	14,249 (73.8)	6,320 (75.4)	661 (66.8)	3,664 (72.3)	1,283 (72.4)	2,381 (72.3)	3,604 (74.0)
Beta-blockers, (%)	13,965 (72.3)	6,215 (74.2)	712 (71.9)	3,433 (67.8)	1,209 (68.2)	2,224 (67.5)	3,605 (74.0)
ACEi/ARB, (%)	10,455 (54.2)	5,069 (60.5)	405 (40.9)	2,044 (40.3)	702 (39.6)	1,342 (40.7)	2,937 (60.3)
Sacubitril-valsartan, (%)	2,156 (11.2)	1,003 (12.0)	97 (9.8)	478 (9.4)	185 (10.4)	293 (8.9)	578 (11.9)
MRA, (%)	3,655 (18.9)	1,598 (19.1)	206 (20.8)	881 (17.4)	314 (17.7)	567 (17.2)	970 (19.9)
SGLT2i, (%)	1,895 (9.8)	783 (9.3)	95 (9.6)	526 (10.4)	189 (10.7)	337 (10.2)	491 (10.1)
Digoxin, (%)	4,481 (23.2)	2,167 (25.9)	204 (20.6)	1,025 (20.2)	368 (20.8)	657 (19.9)	1,085 (22.3)
Ivabradine, (%)	1,471 (7.6)	719 (8.6)	48 (4.9)	306 (6.0)	125 (7.1)	181 (5.5)	398 (8.2)
Hydralazine and nitrate, (%)	135 (0.7)	62 (0.7)	8 (0.8)	30 (0.6)	13 (0.7)	17 (0.5)	35 (0.7)

*ACEi* Angiotensin-converting enzyme inhibitors, *ARB* angiotensin receptor II blockers, *HF* Heart failure, *HFmrEF* Heart failure with mildly reduced ejection fraction, *HFpEF* Heart Failure with preserved ejection fraction, *HFrEF* Heart Failure with reduced ejection fraction, *HFuEF* Heart Failure with unspecified ejection fraction, *MRA* mineralocorticoid receptor antagonists, *SGLT2i* Sodium-glucose co-transporter-2 inhibitors

^a^If the patient had at least a prescription in the 1-year basal period, (s)he was counted in the corresponding medication

**Fig. 1:**
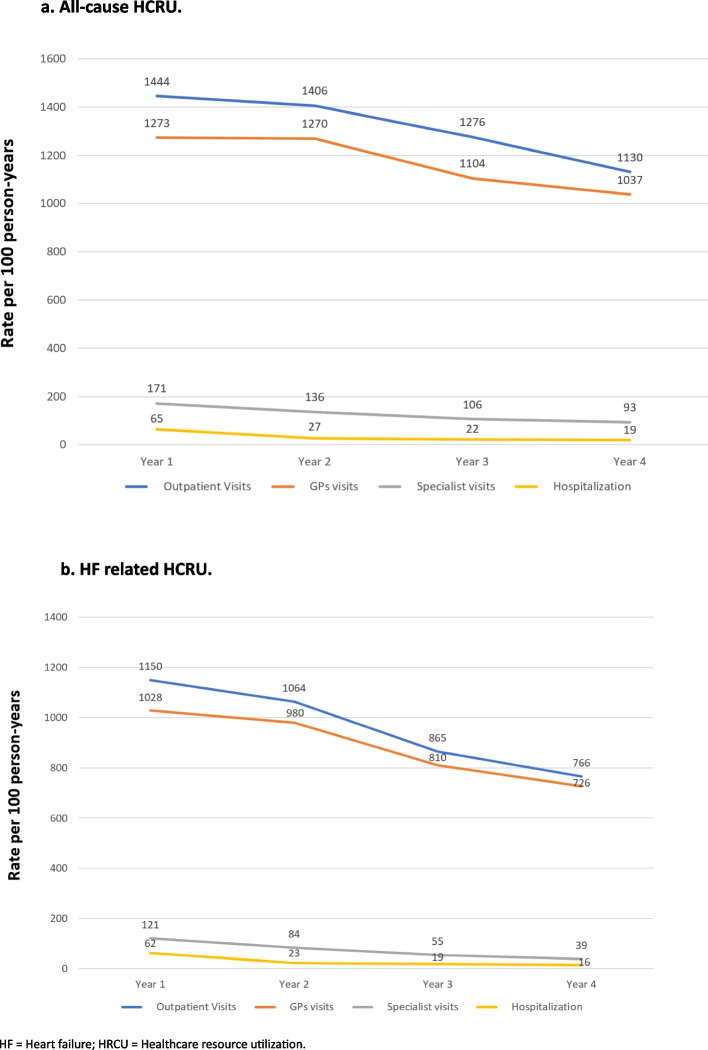
Outpatient visits and hospitalization in the total incident HF Cohort. **a** All-cause HCRU. **b** HF related HCRU

HCRU (outpatient visits and hospitalizations) is shown in Fig. 1a and b, Table 3 and Supplementary Table 2. Overall rates of outpatient visits decreased from 1444 (95% CI 1434.3–1455.3) to 1129.8 (95% CI 1101.1–1156.8) per 100 person-years and hospitalizations from 64.9 (95% CI 64.0–65.9) to 19 (17.9–20.0) per 100 person-years, respectively (Fig. 1a). Additionally, number of days absent from work decreased from 34.9 (21.2) days to 28.5 (18.9) in the HF population (Table 3). The majority of HCRU were HF-related (outpatient visits: 67.8–79.6%; hospitalizations: 82.6–95.1%) (Fig. 1b). HCRU was higher in patients with HFrEF than in patients with HFpEF. Patients with HFmrEF had intermediate HCRU rates. During the follow-up there was a decrease in HCRU, regardless of HF subtypes. Length of hospital stays were longer in patients with HFrEF than in patients with HFpEF, with intermediate values in those patients with HFmrEF. Similarly, work absences and prescriptions were more frequent in the HFrEF population compared to HFpEF patients.

**Table 3:** HCRU in the Incident HF Cohort

	**Year 1 since index (*N* = 19,961)**	**Year 2 since index (*N* = 19,338)**	**Year 3 since index (*N* = 14,381)**	**Year 4 since index (*N* = 9,812)**
**All HF patients**				
**Length of hospital stays (days) (all-cause)**				
Number of patients hospitalized (%)	10,960 (54.9)	4,034 (20.9)	2,463 (17.1)	1,473 (15.0)
Mean (SD)	9.9 (10.6)	12.9 (9.1)	12.0 (7.4)	11.7 (6.9)
Median (IQR)	7 (3—11)	11 (7—18)	11 (6—16)	11 (6—16)
**Length of hospital stays (days) (HF-related)**				
Number of patients hospitalized (%)	10,615 (50.9)	3,898 (20.2)	2,381 (16.6)	1,415 (14.4)
Mean (SD)	9.3 (9.5)	11.1 (8.5)	10.4 (6.9)	10.2 (6.6)
Median (IQR)	7 (4—10)	9 (5—15)	9 (5—15)	9 (5—14)
**Prescriptions (no.) (all-cause)**				
Number of patients (%)	19,961 (100)	19,272 (99.7)	14,176 (98.6)	9,656 (98.4)
Mean (SD)	45.5 (15.6)	40.3 (18.2)	38.3 (19.2)	38.1 (18.9)
Median (IQR)	45 (35—56)	41 (28—53)	39 (25—52)	38 (25—51)
**Prescriptions (no.) (HF-related)**				
Number of patients (%)	19,961 (100)	19,172 (88.8)	14,060 (97.8)	9,583 (97.7)
Mean (SD)	19.5 (9.2)	17.3 (9.6)	16.5 (9.8)	16.4 (9.7)
Median (IQR)	19 (13—25)	17 (10—24)	16 (9—23)	16 (9—23)
**Work absences (days) (all-cause)**				
Number of patients (%)	2,368 (11.9)	2,803 (14.5)	2,204 (15.3)	1,864 (19.0)
Mean (SD)	34.9 (21.2)	38.0 (18.2)	31.0 (19.2)	28.5 (18.9)
Median (IQR)	31 (23—41)	31 (22—45)	29 (21—36)	28 (21—34)
**Work absences (days) (HF-related)**				
Number of patients (%)	2,099 (10.5)	2,492 (12.9)	1,925 (13.4)	1,604 (16.3)
Mean (SD)	28.9 (18.9)	29.7 (18.2)	23.9 (19.2)	21.6 (18.9)
Median (IQR)	27 (19—35)	26 (17—36)	23 (15—31)	22 (16—29)
**HFrEF**				
**Length of hospital stays (days) (all-cause)**				
Number of patients hospitalized (%)	7,927 (39.7)	2,085 (10.8)	1,371 (9.5)	816 (8.3)
Mean (SD)	12.1 (11.3)	16.9 (9.9)	15.3 (7.5)	14.8 (7.0)
Median (IQR)	9 (6—12)	16 (10—22)	15 (9.5—21)	15 (10—20)
**Length of hospital stays (days) (HF-related)**				
Number of patients hospitalized (%)	7,890 (39.5)	2,085 (10.8)	1,371 (9.5)	816 (8.3)
Mean (SD)	10.9 (10.3)	15.2 (9.2)	13.7 (6.9)	13.3 (6.7)
Median (IQR)	8 (5—11)	14 (8—19)	13 (8—18)	13 (8—17)
**Prescriptions (no.) (all-cause)**				
Number of patients (%)	8,678 (43.5)	8,365 (43.3)	6,157 (42.8)	4,290 (43.7)
Mean (SD)	49.5 (15.5)	43.9 (18.9)	42.2 (19.8)	41.1 (19.6)
Median (IQR)	49 (39—60)	44 (32—57)	43 (29—56)	42 (28—55)
**Prescriptions (no.) (HF-related)**				
Number of patients (%)	8,678 (43.5)	8,323 (43.0)	6,115 (42.5)	4,265 (43.5)
Mean (SD)	20.9 (9.2)	18.6 (9.8)	17.9 (10.0)	17.5 (9.9)
Median (IQR)	20 (14—27)	18 (11—25)	17 (10—24)	17 (10—24)
**Work absences (days) (all-cause)**				
Number of patients (%)	1,770 (8.9)	1,916 (9.9)	1,592 (11.1)	1,374 (14.0)
Mean (SD)	38.9 (20.2)	43.5 (24.0)	36.2 (15.6)	32.8 (10.8)
Median (IQR)	34 (28—43)	35 (29—47)	33 (27—41)	31 (26—36)
**Work absences (days) (HF-related)**				
Number of patients (%)	1,636 (8.2)	1,824 (9.4)	1,470 (10.2)	1,262 (12.9)
Mean (SD)	33.6 (18.1)	36.0 (20.1)	29.3 (13.2)	26.1 (9.3)
Median (IQR)	29 (23—37)	29 (24—39)	27 (21—33)	25 (20—30)
**HFmrEF**				
**Length of hospital stays (days) (all-cause)**				
Number of patients hospitalized (%)	370 (1.9)	198 (1.0)	119 (0.8)	64 (0.7)
Mean (SD)	5.7 (6.5)	12.4 (7.5)	10.7 (5.4)	10.8 (5.5)
Median (IQR)	3 (2—7)	11 (7—16)	11 (6—15)	10 (6—15.25)
**Length of hospital stays (days) (HF-related)**				
Number of patients hospitalized (%)	353 (1.8)	197 (1.0)	117 (0.8)	63 (0.6)
Mean (SD)	4.3 (4.8)	9.0 (5.4)	7.9 (4.0)	7.9 (3.8)
Median (IQR)	2 (1—6)	8 (5—11)	8 (5—11)	7 (5—11.5)
**Prescriptions (no.) (all-cause)**				
Number of patients (%)	1,022 (5.1)	986 (5.1)	751 (5.2)	467 (4.8)
Mean (SD)	40.9 (14.1)	37.6 (16.5)	33.5 (17.9)	34.1 (18.0)
Median (IQR)	40 (31—50)	38 (27—48)	33 (20—46)	35 (20—46)
**Prescriptions (no.) (HF-related)**				
Number of patients (%)	1,022 (5.1)	983 (5.1)	747 (5.2)	460 (4.7)
Mean (SD)	17.8 (8.6)	16.4 (9.1)	14.5 (9.2)	14.9 (9.2)
Median (IQR)	17 (11—23)	16 (9—22)	13 (7—21)	14 (7—21)
**Work absences (days) (all-cause)**				
Number of patients (%)	68 (0.3)	101 (0.5)	68 (0.5)	50 (0.5)
Mean (SD)	24.4 (19.0)	30.3 (24.1)	18.7 (8.5)	19.8 (15.1)
Median (IQR)	18 (13—25)	21 (14—29)	16 (13—22.25)	16 (12—23)
**Work absences (days) (HF-related)**				
Number of patients (%)	54 (0.3)	81 (0.4)	46 (0.3)	31 (0.3)
Mean (SD)	16.0 (13.0)	18.3 (14.6)	9.5 (5.0)	8.7 (6.6)
Median (IQR)	11 (9—16.5)	12 (8—29)	8 (6.25—11)	6 (5—9)
**HFpEF**				
**Length of hospital stays (days) (all-cause)**				
Number of patients hospitalized (%)	1,331 (6.7)	869 (4.5)	496 (3.4)	301 (3.1)
Mean (SD)	4.4 (5.6)	9.0 (5.6)	8.4 (4.7)	7.9 (4.6)
Median (IQR)	2 (1—5)	9 (5—12)	9 (4—12)	8 (4—12)
**Length of hospital stays (days) (HF-related)**				
Number of patients hospitalized (%)	1,176 (5.9)	844 (4.4)	481 (3.3)	292 (3.0)
Mean (SD)	2.9 (3.6)	5.5 (3.3)	5.1 (2.7)	4.9 (2.7)
Median (IQR)	1 (1—4)	5 (3—7)	5 (3—7)	5 (3—7)
**Prescriptions (no.) (all-cause)**				
Number of patients (%)	5,244 (26.3)	5,053 (26.1)	3,712 (25.8)	2,562 (26.1)
Mean (SD)	41.1 (14.5)	36.3 (16.7)	34.5 (17.4)	34.5 (17.3)
Median (IQR)	40 (31—51)	36 (25—48)	34 (22—46.25)	35 (22—47)
**Prescriptions (no.) (HF-related)**				
Number of patients (%)	5,244 (26.3)	5,016 (25.9)	3,677 (25.6)	2536 (25.8)
Mean (SD)	17.3 (8.7)	15.4 (9.0)	14.6 (9.0)	14.7 (9.1)
Median (IQR)	16 (11—22)	14 (9—21)	14 (8—20)	14 (8—20)
**Work absences (days) (all-cause)**				
Number of patients (%)	241 (1.2)	380 (2.0)	271 (1.9)	208 (2.1)
Mean (SD)	22.4 (20.5)	26.5 (20.8)	16.3 (9.6)	15.3 (8.8)
Median (IQR)	14 (11—23)	20 (13—26.25)	14 (11—20.5)	13 (10—19)
**Work absences (days) (HF-related)**				
Number of patients (%)	186 (0.9)	290 (1.5)	193 (1.3)	141 (1.4)
Mean (SD)	12.0 (11.1)	12.3 (9.8)	6.1 (4.2)	4.6 (2.9)
Median (IQR)	7 (5—12.75)	9 (6—12.75)	5 (4—7)	4 (3—5)
**HFpEF (EF 50% to < 60%)**				
**Length of hospital stays (days) (all-cause)**				
Number of patients hospitalized (%)	483 (2.4)	312 (1.6)	173 (1.2)	90 (0.9)
Mean (SD)	4.7 (5.7)	8.8 (5.5)	8.3 (5.0)	8.3 (4.7)
Median (IQR)	2 (1—6)	8 (4—13)	9 (4—12)	8.5 (5—11.75)
**Length of hospital stays (days) (HF-related)**				
Number of patients hospitalized (%)	438 (2.2)	305 (1.6)	168 (1.2)	89 (0.9)
Mean (SD)	3.1 (3.6)	5.3 (3.3)	5.1 (2.9)	5.1 (2.8)
Median (IQR)	1 (1—5)	5 (3—7)	5 (2—7)	5 (3—7)
**Prescriptions (no.) (all-cause)**				
Number of patients (%)	1,833 (9.2)	1,770 (9.2)	1,316 (9.2)	889 (9.1)
Mean (SD)	40.5 (14.5)	35.8 (16.5)	33.3 (16.9)	33.4 (17.0)
Median (IQR)	40 (31—49)	36 (25—47)	34 (21—45)	34 (21—46)
**Prescriptions (no.) (HF-related)**				
Number of patients (%)	1,833 (9.5)	1,757 (9.1)	1,302 (9.1)	883 (9.0)
Mean (SD)	17.1 (8.7)	15.3 (9.0)	14.2 (8.8)	14.4 (9.1)
Median (IQR)	16 (11—22)	14 (9—21)	13 (7—20)	13 (7—21)
**Work absences (days) (all-cause)**				
Number of patients (%)	85 (0.4)	143 (0.7)	87 (0.6)	66 (0.7)
Mean (SD)	25.4 (22.7)	28.9 (23.5)	16.9 (10.6)	15.6 (11.2)
Median (IQR)	17 (11—25)	20 (13—27)	14 (11—21)	12.5 (11—17.75)
**Work absences (days) (HF-related)**				
Number of patients (%)	67 (0.3)	111 (0.6)	59 (0.4)	45 (0.5)
Mean (SD)	13.8 (12.1)	13.4 (10.9)	6.3 (5.1)	4.8 (3.6)
Median (IQR)	8 (6—14)	9 (6—22)	5 (4—7)	4 (3—6)
**HFpEF (EF ≥ 60%)**				
**Length of hospital stays (days) (all-cause)**				
Number of patients hospitalized (%)	848 (4.2)	557 (2.9)	323 (2.2)	211 (2.2)
Mean (SD)	4.2 (5.5)	9.1 (5.6)	8.4 (4.5)	7.8 (4.6)
Median (IQR)	2 (1—4)	9 (5—12)	9 (5—12)	7 (4—11.5)
**Length of hospital stays (days) (HF-related)**				
Number of patients hospitalized (%)	738 (3.7)	539 (2.8)	313 (2.2)	203 (2.1)
Mean (SD)	2.8 (3.5)	5.6 (3.3)	5.1 (2.6)	4.8 (2.7)
Median (IQR)	1 (1—3)	5 (3—7.5)	5 (3—7)	5 (3—7)
**Prescriptions (no.) (all-cause)**				
Number of patients (%)	3,411 (17.1)	3,283 (17.0)	2,396 (16.7)	1,673 (17.1)
Mean (SD)	41.3 (14.5)	36.5 (16.8)	35.1 (17.6)	35.0 (17.5)
Median (IQR)	41 (31—51)	37 (25—48)	35 (22.75—47)	35 (23—47)
**Prescriptions (no.) (HF-related)**				
Number of patients (%)	3,411 (17.1)	3,259 (16.9)	2,375 (16.5)	1,653 (16.8)
Mean (SD)	17.3 (8.7)	15.4 (9.0)	14.8 (9.1)	14.9 (9.1)
Median (IQR)	16 (11—23)	14 (8—21)	14 (8—20.5)	14 (8—20)
**Work absences (days) (all-cause)**				
Number of patients (%)	156 (0.8)	237 (1.2)	184 (1.3)	142 (1.4)
Mean (SD)	20.7 (19.0)	25.0 (18.9)	16.0 (9.0)	15.2 (7.4)
Median (IQR)	13 (11—22.25)	20 (13—26)	13 (10—20)	13 (10—19)
**Work absences (days) (HF-related)**				
Number of patients (%)	119 (0.6)	179 (0.9)	134 (0.9)	96 (1.0)
Mean (SD)	11.0 (10.4)	11.7 (8.9)	6.0 (3.8)	4.5 (2.5)
Median (IQR)	6 (5—11)	9 (6—12)	5 (4—7)	4 (3—5)
**HFuEF**				
**Length of hospital stays (days) (all-cause)**				
Number of patients hospitalized (%)	1,332 (6.7)	882 (4.6)	477 (3.3)	292 (3.0)
Mean (SD)	4.1 (4.9)	7.5 (4.5)	6.4 (3.8)	6.9 (3.7)
Median (IQR)	2 (1—5)	7 (4—10)	6 (3—9)	7 (4—10)
**Length of hospital stays (days) (HF-related)**				
Number of patients hospitalized (%)	1,196 (6.0)	772 (4.0)	412 (2.9)	244 (2.5)
Mean (SD)	6.8 (3.9)	6.9 (4.1)	6.5 (3.5)	6.9 (3.6)
Median (IQR)	6 (4—10)	7 (4—10)	7 (3—9)	7 (4—10)
**Prescriptions (no.) (all-cause)**				
Number of patients (%)	5,017 (25.1)	4,868 (25.2)	3,556 (24.7)	2337 (23.8)
Mean (SD)	44.3 (15.3)	39.1 (17.8)	36.6 (18.9)	37.4 (18.5)
Median (IQR)	44 (34—54)	39 (27—51)	37 (23—50)	38 (24—50)
**Prescriptions (no.) (HF-related)**				
Number of patients (%)	5,017 (25.1)	4,850 (25.1)	3,521 (24.5)	2,322 (23.7)
Mean (SD)	19.6 (9.2)	17.3 (9.6)	16.3 (9.9)	16.6 (9.8)
Median (IQR)	19 (13—26)	16 (10—24)	15 (8—23)	16 (9—23)
**Work absences (days) (all-cause)**				
Number of patients (%)	289 (1.4)	406 (2.1)	273 (1.9)	232 (2.4)
Mean (SD)	23.3 (19.3)	24.8 (19.0)	17.8 (9.3)	16.7 (7.5)
Median (IQR)	16 (13—22)	17 (15—24)	16 (13—20)	15 (13—19)
**Work absences (days) (HF-related)**				
Number of patients (%)	223 (1.1)	297 (1.5)	216 (1.5)	170 (1.7)
Mean (SD)	11.5 (9.9)	11.1 (8.8)	6.3 (3.6)	4.9 (2.4)
Median (IQR)	8 (6—10)	8 (6—11)	6 (5—7)	4 (3—6)

IQR must be 25th and 75th percentiles; length of hospital stays and work absences are expressed in days and prescriptions in pills

*HF* Heart failure, *HFmrEF* Heart failure with mildly reduced ejection fraction, *HFpEF* Heart Failure with preserved ejection fraction, *HFrEF* Heart failure with reduced ejection fraction, *HFuEF* Heart Failure with unspecified ejection, *HRCU* Healthcare resource utilization, *IQR* Interquartile range, *SD* Standard deviation

**Fig. 2:**
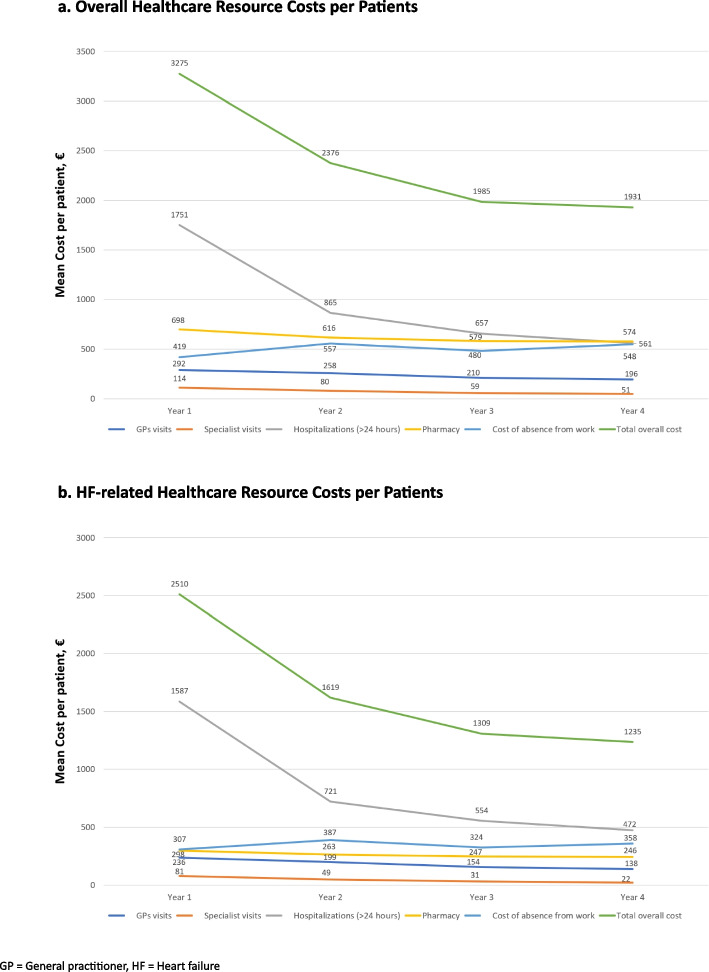
Healthcare resource costs per patients by years since index in the total Incident HF cohort. **a **Overall healthcare resource costs per patients. **b **HF-related healthcare resource costs per patients

Overall and HF-related healthcare resource costs per patient by years since index date are shown in Figs. 2a and b and Supplementary Tables 3 and 4. Total mean (SD) per patient overall cost decreased from 3274.7 (4053.4) to 1931.2 (1949.1) Euros (41% cost reduction) (Fig. 2a), and mean (SD) per patient HF-related costs decreased from 2509.6 (3518.5) to 1234.6 (1534.1) Euros (50% cost reduction) (Fig. 2b). When considering overall healthcare resource costs per patient in the 4 years after HF diagnosis, the majority of costs were due to hospitalizations (year 1: 53.5%; year 4: 29.1%), followed by pharmacy (year 1: 21.3%; year 4: 29.7%), indirect costs (year 1: 12.8%; year 4: 28.4%), and outpatient care (year 1: 12.4%; year 4: 12.8%). When analyzing HF-related healthcare resource costs per patient in the same 4 years, the majority of costs were due to hospitalizations (year 1: range: 63.3%; year 4: 38.2%), followed by indirect costs (year 1: 12.2%; year 4: 29.0%), pharmacy (year 1: 11.9%; year 4: 19.9%), and outpatient care (year 1: 12.6%; year 4: 12.9%). Overall and HF-related healthcare resource costs per patient were greater in patients with HFrEF compared to patients with HFpEF, with intermediate values for patients with HFmrEF.

## Discussion

This study showed that in Spain economic burden of patients with HF was high. HCRU and costs decreased over time since diagnosis, in part possibly due to a survival bias, but also due to the use of HF therapies. Despite that, there is still much room for improvement and a higher use of guideline-recommended therapies would be desirable.

This study, used data from electronic health records of nearly two million people who were fully anonymized and integrated. A number of studies have shown the value of the secondary use of electronic health record datasets to provide relevant information about the evolution and management of the HF population and other chronic disease [21–24]. We have endeavored to add to that literature using the BIG-PAC database in Spain which has been demonstrated to have validity and representativeness in assessing clinical profiles, management and healthcare costs of HF in Spain [1, 6, 11, 18].

In nearly 20,000 patients with newly diagnosed HF (de novo HF), around 44% of patients had HFrEF and 26% HFpEF (58% and 25%, respectively if we consider only those with known EF). The relative proportions of the EF phenotypes in patients with HF varies considerably across countries and across studies. Thus, in a study performed in China, 57% of patients had HFpEF, 21% HFmrEF, and 22% HFrEF [3]. In a study performed in US, 30.8% had HFpEF, 45.9% HFrEF, and the remaining 23.3% were unspecified in terms of HF type [4]. In a study performed in Sweeden, 64.5% of patients had HFrEF and 35.5% HFpEF [14]. In other study performed in Spain, the proportion of patients among different HF subtypes clearly depending on the specialty that attended patients (i.e., internal medicine vs cardiology) [25]. These differences can be partly attributable to the proportion of patients with unknown EF in each study, the age of their patients, and the clinical setting in which patients are attended [3, 4, 12, 14, 25, 26]. However, as the BIG-PAC database collected information from electronic medical records of both inpatient and outpatient settings from hospital specialties and primary care in seven different Autonomous Communities in Spain, the information provided was balanced, comprehensive and with a lower risk of bias, supporting that the results can be extended to the whole Spanish population [1, 11, 18].

With regard to the use of disease modifying HF drugs, at 12 months after initial HF diagnosis, 72% of patients were taking beta blockers, 67% renin-angiotensin system inhibitors (11% sacubitril-valsartan), 19% mineralocorticoid receptor antagonists and 10% SGLT2i (74%, 72.5% -12.0%-, 19%, and 9% among patients with HFrEF, respectively). Although it should be considered that recommendations from ESC guidelines have changed within the study period (i.e., current ESC guidelines for treatment of HF did not include SGLT2i until late in our study period), our data showed that a higher use of guideline-recommended therapies would be desirable [7, 27]. The PARADIGM-HF trial of sacubitril-valsartan was published in 2014 [28] and clinical trials showing the benefits of SGLT2i in both patients with HFrEF and HFpEF were published later. Our study extends to a time before these clinical trials and the most recent guidelines [28–34], so we would not expect the uptake of new guidelines to be evident in our results. However, as these drugs have demonstrated robust clinical benefits over traditional approach, their use should be highly promoted, as current European guidelines recommend [7].

We found a marked decrease in the use of healthcare resources over a 4-year follow-up period since HF diagnosis, including outpatient care and hospitalizations, particularly for HF hospitalizations (from 61.7 to 15.7 per 100 person-years). Some of this decrease may be attributable to a survival bias, in which healthier patients who use fewer resources survive longer than sicker patients or with a delayed care/diagnosis and this could explain some of the differences found with other studies, with different methodology and HF population (i.e., incident vs prevalent cohorts, outpatient vs in hospital patients, differences in clinical profile, etc.) [35, 36]. But there are other factors that may contribute to this trend. First, the awareness of HF has increased, in part, due to the recent availability of new, more effective treatments such as sacubitril-valsartan and SGLT2i—leading to an improvement of the early diagnosis and initiation of HF treatment [37]. It has been demonstrated that time to initiation of disease modifying therapies is an important clinical target. Rapid introduction and up-titration of therapy and introduction early in the disease progression have been shown to have clinical benefits [38]. In addition, general practitioners play a key role in the diagnosis and chronic management of this population. Our study showed that mean number of HF-related general practitioner visits per patients was higher than for specialist visits, providing more opportunity for monitoring and management. Different studies have demonstrated that a better coordination between different healthcare levels reduces not only hospitalization, but also mortality among patients with HF [39, 40]. Despite that, the risk of readmissions following a HF hospitalization or visit to the emergency department due to HF decompensation is still high [12, 41]. Delaying medical care is associated with a higher HF burden, including medical costs [38, 42].

We looked at work absences, which are available in this healthcare database, as a way to assess the indirect costs associated with HF. Work absences only represent part of the indirect costs of HF as mean age of the HF population was 70 years; beyond usual working age. Indirect costs include loss of work, but may also include informal caregiving, which may be similar or greater than the direct health care costs [8]. As a result, particular attention should be paid on this issue and should be included in future research.

With regard to HF-related healthcare costs per patient, HF hospitalizations were the most important determinant of total cost, followed by indirect costs, pharmacy and outpatient care. Of note, HF-related healthcare costs decreased by 50% after a 4-year period of follow-up. However, this reduction was not homogeneous, as there was a marked decrease of costs related to hospitalizations and outpatient care, whereas costs related to pharmacy only showed a small decrease and indirect costs increased over time. This means that to actually reduce HF cost burden, it is necessary to reduce HF hospitalizations, particularly among high-risk patients, such as those with a recent worsening HF. Not only the early implementation of disease modifying HF treatment is important, but also improving the coordination between different healthcare levels [9, 14, 39, 43, 44]. This would facilitate the optimization of the management of patients with HF [44, 45]. In fact, improving adherence to guideline-recommended therapy is associated with a reduction of healthcare costs [39, 44, 46]. In this context, those drugs that provide benefits on HF hospitalizations, could be more cost-effective [47]. The costs related to pharmacy also showed a small reduction during follow-up. Since HF hospitalizations are the main driver of costs, those drugs that reduce these outcomes may translate into a reduction of healthcare costs. Thus, recent studies have shown that the addition of SGLT2i to standard therapy represents a highly cost-effective approach [48–50]. In other words, delaying the prescription of disease modifying HF drugs is associated with a higher risk of unwanted events and increased healthcare costs [38, 42]. In our study, indirect costs increased during the study period. Optimization of treatment after first diagnosis could help not only to reduce the length of inpatient stays, and the number of outpatient visits, but periods of disability that contribute to work absence [51, 52].

Finally, our study showed that overall and HF-related healthcare resource use and costs per patient were greater in patients with HFrEF compared to other HF subtypes. Not only are there relevant differences in the clinical profile of patients and the prescription of HF drugs across EF phenotypes, but as other authors have reported, also the use of invasive diagnostic and therapeutic procedures is more common in patients with HFrEF [1, 44, 45]. Previous studies have shown an inverse relationship between EF and costs, with more invasive procedures and more readmissions in patients with lower EF [22, 23, 53].

Due to the retrospective study design, only those variables recorded in the medical history of patients could be used. In addition, discounting costs or no sensitivity analysis for price ranges was not considered. Moreover, HF treatments were recorded at baseline and during 12 months of follow-up from diagnosis, but not thereafter. However, the large number of patients included in the study mitigates these limitations. In addition, as costs directly depend on the healthcare system characteristics, these results can be extended only to those locations in which patients with HF have a similar clinical profile and healthcare management.

In conclusion, the HCRU and economic burden of HF were highest in the initial period after first diagnosis for all EF phenotypes, owing largely to high inpatient costs. Despite costs decreased over time, they remained high. The observed decline could be related with survival bias, but also to a better management of patients with HF, including a higher proportion of patients receiving disease modifying HF drugs. Despite that, there is still room for improvement in the identification and treatment of patients with HF early in disease progression, as recommended in current guidance, when initial hospitalizations and readmissions are more common, and high costs could substantially be reduced.

### Supplementary Information

**Additional file 1: Supplementary Table 1.** Description of costs/units (2019)*. **Supplementary Table 2.** Outpatient visits and hospitalization in the incident HF Cohort. **Supplementary Table 3.** Overall healthcare resource costs per patients by years since index in the incident HF cohort. **Supplementary Table 4.** HF-related healthcare resource costs per patients by years since index in the incident HF cohort.

## Data Availability

This was a secondary data study using the BIG-PAC® database, and the data can be obtained upon reasonable request to Atrys Health S.A. (Ignacio Hernández. E-mail: ihernandez@atryshealth.com).
