# Supplementary material for: Evolution of economic burden of heart failure by ejection fraction in newly diagnosed patients in Spain
Source: BMC Health Serv Res. 2023 Dec 1;23:1340. doi: 10.1186/s12913-023-10376-z (PMC10693147; doi:10.1186/s12913-023-10376-z)
Supplement: Supplementary file 1 — Additional file 1: Supplementary Table 1. Description of costs/units (2019)*. Supplementary Table 2. Outpatient visits and hospitalization in the incident HF Cohort. Supplementary Table 3. Overall healthcare resource costs per patients by years since index in the incident HF cohort. Supplementary Table 4. HF-related healthcare resource costs per patients by years since index in the incident HF cohort. [file 12913_2023_10376_MOESM1_ESM.docx]

**Supplementary table 1. Description of costs/units (2019)***

| **Health and non-health resources** | **Unit costs (A)** |
| --- | --- |
| Medical visits |  |
| Medical visits and primary care | €24.20 |
| Emergency room visit | €118.50 |
| Hospitalization (per day) | €480.90 |
| Specialist medical visit | €94.50 |
| Supplementary tests |  |
| Lab tests | €32.30 |
| Conventional radiology | €28.50 |
| Diagnostic test/therapeutic | €37.10 |
| Computerized axial tomography | €96.00 |
| Magnetic nuclear resonance | €177.00 |
| Pharmaceutical prescription | RP+VAT |
| Work productivity - Indirect costs |  |
| Cost per day not worked | €101.21 |

*Costs were considered as by the year 2019. Values are expressed in euros

RP: retail price; VAT: value-added tax

**Supplementary Table 2. Outpatient Visits and Hospitalization in the Incident HF Cohort.**

|  | **Year 1 after Index Date (n=19,961)** | | | | **Year 2 after Index Date (n=19,338)** | | | | **Year 3 after Index Date (n=14,381)** | | | | **Year 4 after Index Date (n=9,812)** | | | | |  |
| --- | --- | --- | --- | --- | --- | --- | --- | --- | --- | --- | --- | --- | --- | --- | --- | --- | --- | --- |
|  | **Number of patients** | **Number of visits** | **Total person-time in years** | **Rate per 100 person-years (95% CI)** | **Number of patients** | **Number of visits** | **Total person-time in days** | **Rate per 100 person-years (95% CI)** | **Number of patients** | **Number of visits** | **Total person-time in days** | **Rate per 100 person-years (95% CI)** | | **Number of patients** | **Number of visits** | **Total person-time in days** | **Rate per 100 person-years (95% CI)** | |
| **All HF patients** | | | | | | | | | | | | | | | | | | |
| **All-cause HCRU** | | | | | | | | | | | | | | | | | | |
| Outpatient Visits | 19,452 | 285,351 | 19,761 | 1,444.0 (,1434.3-1,455.3) | 18,838 | 23,7813 | 16,914 | 1,406.0 (1,390.8-1,422.1) | 13,625 | 15,0598 | 1,1802 | 1,276.1 (1,252.1-1,297.9) | | 9,347 | 90,459 | 8,007 | 1,129.8 (1,101.1-1,156.8) | |
| GPs visits | 19,031 | 251,565 | 19,761 | 1,273.0 (1,263.7-1,283.2) | 18,576 | 21,4813 | 16,914 | 1,270.0 (1,256.0-1,284.8) | 13,613 | 13,0274 | 1,1802 | 1,103.9 (1,083.1-1,122.8) | | 9,252 | 83,018 | 8,007 | 1,036.8 (1,009.9-1,062.3) | |
| Specialist visits | 12,090 | 33,786 | 19,761 | 1,71.0 (168.7-173.4) | 8,494 | 23,000 | 16,914 | 136.0 (133.2-138.6) | 4,696 | 1,2549 | 1,1802 | 106.3 (103.2-109.6) | | 2,809 | 7,441 | 8,007 | 92.9 (88.9-96.86) | |
| Hospitalization | 10,960 | 12,826 | 19,761 | 64.9 (64.0-65.9) | 4,034 | 4,635 | 16,914 | 27.4 (26.6-28.2) | 2,463 | 2578 | 1,1802 | 21.8 (21.0-22.7) | | 1,473 | 1,518 | 8,007 | 19.0 (17.9-20.0) | |
| **HF-related HCRU** | | | | | | | | | | | | | | | | | | |
| Outpatient Visits | 19,390 | 227,144 | 19,761 | 1,149.5 (1,140.8-1,159.3) | 18,732 | 17,9915 | 16,914 | 1,063.7 (1,051.9-1,076.3) | 13,608 | 102,099 | 1,1802 | 865.1 (848.6-880.8) | | 9,220 | 61,295 | 8,007 | 765.5 (745.9-784.5) | |
| GPs visits | 19,020 | 203,201 | 19,761 | 1,028.3 (1,020.3-1,037.1) | 18,507 | 16,5774 | 16,914 | 980.1 (969.3-991.4) | 13,502 | 95,571 | 1,1802 | 809.8 (794.3-824.4) | | 9,190 | 58,163 | 8,007 | 726.4 (707.6-744.2) | |
| Specialist visits | 11,347 | 23,943 | 19,761 | 121.2 (119.3–123.0) | 7431 | 1,4141 | 16,914 | 83.6 (81.6-85.4) | 3,671 | 6,528 | 1,1802 | 55.3 (53.4-57.4) | | 1,802 | 3,132 | 8,007 | 39.1 (37.1-41.1) | |
| Hospitalization | 10,675 | 12,197 | 19,761 | 61.7 (60.9-62.7) | 3530 | 3832 | 16,914 | 22.7 (21.9-23.4) | 2,128 | 2,179 | 1,1802 | 18.5 (17.7-19.2) | | 1,233 | 1,258 | 8,007 | 15.7 (14.7-16.7) | |
| **HFrEF** | | | | | | | | | | | | | | | | | | |
| **All-cause HCRU** | | | | | | | | | | | | | | | | | | |
| Outpatient Visits | 8,678 | 183,127 | 8,584 | 2,133.4 (2,121.4-2,147.0) | 8,350 | 156,009 | 7,335 | 2,126.8 (2,101.7-2,151.0) | 6,127 | 10,1322 | 5,182 | 1,955.4 (1,908.4-2,001.8) | | 4,272 | 5,8414 | 3,537 | 1,651.7 (1,592.9-1,707.3) | |
| GPs visits | 8,674 | 162,795 | 8,584 | 1,896.5 (1,885.0 1,909.1) | 8,325 | 141,282 | 7,335 | 1,926.0 (1,901.7-1,948.8) | 6,115 | 8,2106 | 5,182 | 1,584.6 (1,545.6-1,623.8) | | 4,254 | 5,3771 | 3,537 | 1,520.4 (1,464.6-1,573.5) | |
| Specialist visits | 6,773 | 20,332 | 8,584 | 236.9 (233.2-240.8) | 5,007 | 14,727 | 7,335 | 200.8 (195.7-205.3) | 2,781 | 8,054 | 5,182 | 155.4 (149.0-161.7) | | 1,612 | 4,643 | 3,537 | 131.3 (123.5-138.5) | |
| Hospitalization | 7,927 | 9,468 | 8,584 | 110.3 (109.2-111.5) | 2,085 | 2,473 | 7,335 | 33.7 (32.3-35.1) | 1,371 | 1,466 | 5,182 | 28.3 (26.8-29.8) | | 816 | 850 | 3,537 | 24.0 (22.4-25.7) | |
| **HF-related HCRU** | | | | | | | | | | | | | | | | | | |
| Outpatient Visits | 8,674 | 133,602 | 8,584 | 1,755.3 (1,744.7-1767.4) | 8,346 | 121,436 | 7,335 | 1,655.5 (1,635.8-1,674.3) | 6,138 | 66,318 | 5,182 | 1,279.9 (1,247.8-1,310.8) | | 4,267 | 41,083 | 3,537 | 1,161.6 (1,119.5-1,201.6) | |
| GPs visits | 8,678 | 150,668 | 8,584 | 1,556.5 (1,546.2-1,567.7) | 8,318 | 110,179 | 7,335 | 1,502.0 (1,483.3-1,520.1) | 6,103 | 60,723 | 5,182 | 1,171.9 (1,142.6-1,199.7) | | 4,248 | 38,184 | 3,537 | 1,079.7 (1,040.3-1,118.0) | |
| Specialist visits | 6,658 | 17,066 | 8,584 | 198.8 (195.5- 202.1) | 4,906 | 1,1257 | 7,335 | 153.5 (149.6-156.9) | 2738 | 5,595 | 5,182 | 108.0 (103.6-112.2) | | 1,569 | 2,899 | 3,537 | 82.0 (77.1-86.3) | |
| Hospitalization | 7,915 | 9,305 | 8,584 | 108.4 (107.3-109.5) | 1,837 | 2,028 | 7,335 | 27.7 (26.4-28.8) | 1183 | 1,227 | 5,182 | 23.7 (22.4 - 25.1) | | 683 | 703 | 3,537 | 19.9 (18.3-21.4) | |
| **HFmrEF** | | | | | | | | | | | | | | | | | | |
| **All-cause HCRU** | | | | | | | | | | | | | | | | | | |
| Outpatient Visits | 986 | 9,918 | 1,013 | 979.4 (953.6-1,004.1) | 942 | 7,657 | 892 | 858.9 (829.2-889.3) | 688 | 4,291 | 599 | 715.9 (663.7-763.8) | | 452 | 2,695 | 378 | 712.9 (629.1-789.4) | |
| GPs visits | 945 | 8,559 | 1,013 | 845.2 (822.8-868.6) | 927 | 6,795 | 892 | 762.2 (736.5-788.1) | 688 | 4,133 | 599 | 689.5 (639.2-736.2) | | 440 | 2,417 | 378 | 639.4 (563.4-709.7) | |
| Specialist visits | 529 | 1,359 | 1,013 | 134.2 (124.7-143.7) | 353 | 862 | 892 | 96.7 (86.8-105.0) | 205 | 448 | 599 | 74.7 (63.8- 85.4) | | 121 | 278 | 378 | 73.5 (58.3-87.6) | |
| Hospitalization | 370 | 411 | 1,013 | 40.6 (37.1-44.2) | 198 | 223 | 892 | 25.0 (21.5- 28.0) | 119 | 121 | 599 | 20.2 (16.3-23.3) | | 64 | 67 | 378 | 17.7 (13.3-22.1) | |
| **HF-related HCRU** | | | | | | | | | | | | | | | | | | |
| Outpatient Visits | 981 | 7,579 | 1,013 | 748.4 (727.6-768.1) | 935 | 5,565 | 892 | 624.2 (602.7-647.2) | 686 | 3,178 | 599 | 530.2 (492.6-566.0) | | 437 | 1,738 | 378 | 459.8 (406.1-511.2) | |
| GPs visits | 943 | 6,804 | 1,013 | 671.9 (652.2 691.2) | 923 | 5,243 | 892 | 588.1 (567.6-609.6) | 678 | 3,082 | 599 | 514.2 (476.8-549.2) | | 435 | 1,703 | 378 | 450.5 (395.7-500.8) | |
| Specialist visits | 481 | 775 | 1,013 | 76.5 (70.9-82.2) | 264 | 322 | 892 | 36.1 (32.1- 39.8) | 96 | 96 | 599 | 16.0 (12.7-19.2) | | 35 | 35 | 378 | 9.3 (6.2-12.2) | |
| Hospitalization | 350 | 372 | 1,013 | 36.7 (33.5-40.0) | 172 | 183 | 892 | 20.5 (17.5-23.2) | 103 | 105 | 599 | 17.5 (14.0-20.6) | | 54 | 55 | 378 | 14.6 (10.5-18.3) | |
| **HFpEF** | | | | | | | | | | | | | | | | | | |
| **All-cause HCRU** | | | | | | | | | | | | | | | | | | |
| Outpatient Visits | 4,969 | 45,314 | 5,191 | 873.0 (862.5-883.5) | 4,844 | 39,445 | 4425 | 891.3 (875.0-906.4) | 3,468 | 22,351 | 3,108 | 719.2 (695.85 - 742.27) | | 2,393 | 15,382 | 2,143 | 717.6 (684.6-751.3) | |
| GPs visits | 4,746 | 39,002 | 5,191 | 751.4 (741.6-761.0) | 4,700 | 35,438 | 4425 | 800.8 (786.4-814.2) | 3,468 | 21,910 | 3,108 | 705.0 (681.66 - 727.74) | | 2,357 | 14,076 | 2,143 | 656.7 (626.2-686.5) | |
| Specialist visits | 2,515 | 6,312 | 5,191 | 121.6 (117.6-125.3) | 1,673 | 4,007 | 4425 | 90.6 (86.2-94.9) | 867 | 2,059 | 3,108 | 66.3 (61.72 - 71.03) | | 559 | 1,306 | 2,143 | 60.9 (55.1-66.6) | |
| Hospitalization | 1,331 | 1,472 | 5,191 | 28.4 (27.0-29.8) | 869 | 972 | 4425 | 22.0 (20.5-23.3) | 496 | 505 | 3,108 | 16.3 (14.73 - 17.53) | | 301 | 306 | 2,143 | 14.3 (12.6-15.9) | |
| **HF-related HCRU** |  |  |  |  |  |  |  |  |  |  |  |  | |  |  |  |  | |
| Outpatient Visits | 4,934 | 33,983 | 5,191 | 654.7 (646.5-662.7) | 4,790 | 28,276 | 4425 | 639.0 (627.6-649.9) | 3,442 | 16,335 | 3,108 | 525.6 (508.6-542.4) | | 2,342 | 9,744 | 2,143 | 454.6 (432.2-475.5) | |
| GPs visits | 4,738 | 30,795 | 5,191 | 593.3 (585.3-601.3) | 4,675 | 26,865 | 4425 | 607.1 (595.9-617.8) | 3,412 | 15,895 | 3,108 | 511.4 (494.8-527.5) | | 2,333 | 9,614 | 2,143 | 448.5 (426.2-469.6) | |
| Specialist visits | 2,199 | 3,188 | 5,191 | 61.4 (59.3-63.5) | 1,227 | 1,411 | 4425 | 31.9 (30.2-33.6) | 440 | 440 | 3,108 | 14.2 (12.9-15.4) | | 130 | 130 | 2,143 | 6.1 (4.9-7.1) | |
| Hospitalization | 1,213 | 1,259 | 5,191 | 24.3 (23.0-25.5) | 749 | 806 | 4425 | 18.2 (17.0 - 19.4) | 430 | 433 | 3,108 | 13.9 (12.6-15.2) | | 252 | 254 | 2,143 | 11.9 (10.4-13.3) | |
| **HFpEF (EF 50% to <60%)** | | | | | | | | | | | | | | | | | | |
| **All-cause HCRU** | | | | | | | | | | | | | | | | | | |
| Outpatient Visits | 1,736 | 15,875 | 1,815 | 874.7 (856.6-893.2) | 1,691 | 1,4013 | 1,557 | 900.0 (874.6-925.2) | 1239 | 7885 | 1,085 | 726.4 (686.5-767.6) | | 830 | 5,375 | 735 | 731.4 (673.5-788.0) | |
| GPs visits | 1,657 | 13,650 | 1,815 | 752.1 (735.3-769.3) | 1,655 | 1,2627 | 1,557 | 811.0 (787.2-835.8) | 1239 | 7719 | 1,085 | 711.1 (671.1-751.2) | | 814 | 4,881 | 735 | 664.2 (609.6-715.1) | |
| Specialist visits | 887 | 2,225 | 1,815 | 122.6 (116.0-128.9) | 571 | 1,386 | 1,557 | 89.0 (82.3-96.3) | 320 | 763 | 1,085 | 70.3 (62.5-78.5) | | 209 | 494 | 735 | 67.2 (57.4-77.5) | |
| Hospitalization | 483 | 544 | 1,815 | 30.0 (27.6-32.4) | 312 | 356 | 1,557 | 22.9 (20.3-25.3) | 173 | 177 | 1,085 | 16.3 (14.1-18.8) | | 90 | 92 | 735 | 12.5 (9.8-15.1) | |
| **HF-related HCRU** | | | | | | | | | | | | | | | | | | |
| Outpatient Visits | 1,721 | 11,894 | 1,815 | 655.4 (640.9-671.0) | 1,678 | 10,056 | 1,557 | 645.9 (627.6-665.6) | 1,228 | 5,756 | 1,085 | 530.3 (500.4-561.0) | | 812 | 3,401 | 735 | 462.8 (426.2-499.1) | |
| GPs visits | 1,653 | 10,757 | 1,815 | 592.7 (578.6-606.6) | 1,646 | 9,568 | 1,557 | 614.6 (596.8-634.0) | 1,217 | 5,589 | 1,085 | 514.9 (486.0 -544.2) | | 809 | 3,354 | 735 | 456.4 (419.0-492.4) | |
| Specialist visits | 780 | 1,137 | 1,815 | 62.7 (59.0-66.0) | 421 | 488 | 1,557 | 31.3 (28.5- 34.2) | 167 | 167 | 1,085 | 15.4 (13.2-17.6) | | 47 | 47 | 735 | 6.4 (4.5-8.1) | |
| Hospitalization | 435 | 452 | 1,815 | 24.9 (22.7- 26.9) | 275 | 303 | 1,557 | 19.5 (17.1-21.7) | 150 | 152 | 1,085 | 14.0 (12.1-16.2) | | 82 | 83 | 735 | 11.3 (8.7-13.7) | |
| **HFpEF (EF ≥60%)** | | | | | | | | | | | | | | | | | | |
| **All-cause HCRU** | | | | | | | | | | | | | | | | | | |
| Outpatient Visits | 3,233 | 29,439 | 3,376 | 872.1 (859.9-885.9) | 3,153 | 25,432 | 2,868 | 886.6 (867.3-904.6) | 2229 | 14,466 | 2,022 | 715.3 (685.1-742.4) | | 1,563 | 10,007 | 1,409 | 710.4 (665.2-753.5) | |
| GPs visits | 3,089 | 25,352 | 3,376 | 751.0 (740.2-763.4) | 3,045 | 22,811 | 2,868 | 795.2 (776.7-812.1) | 2229 | 14,191 | 2,022 | 701.7 (672.6-728.3) | | 1,543 | 9,195 | 1,409 | 652.8 (610.52 - 692.09) | |
| Specialist visits | 1,628 | 4087 | 3,376 | 121.1 (116.1-126.2) | 1,102 | 2,621 | 2,868 | 91.4 (86.1-96.3) | 547 | 1,296 | 2,022 | 64.1 (58.3-69.4) | | 350 | 812 | 1,409 | 57.7 (51.2-641) | |
| Hospitalization | 848 | 928 | 3,376 | 27.5 (25.6-29.1) | 557 | 616 | 2,868 | 21.5 (19.7-23.2) | 323 | 328 | 2,022 | 16.2 (14.4-17.8) | | 211 | 214 | 1,409 | 15.2 (13.2-17.3) | |
| **HF-related HCRU** | | | | | | | | | | | | | | | | | | |
| Outpatient Visits | 3,213 | 22,089 | 3,376 | 654.4 (644.7-664.7) | 3,112 | 18,220 | 2,868 | 635.2 (620.7-648.9) | 2,214 | 10,579 | 2,022 | 523.1 (501.4-543.6) | | 1,530 | 6,343 | 1,409 | 450.3 (421.7-477.9) | |
| GPs visits | 3,085 | 20,038 | 3,376 | 593.6 (584.2-603.6) | 3,029 | 17,297 | 2,868 | 603.0 (588.8-616.3) | 2,195 | 10,306 | 2,022 | 509.6 (487.7-529.6) | | 1,524 | 6,260 | 1,409 | 444.4 (416.6-471.7) | |
| Specialist visits | 1,419 | 2,051 | 3,376 | 60.8 (58.1-63.5) | 806 | 923 | 2,868 | 32.2 (30.0-34.3) | 273 | 273 | 2,022 | 13.5 (11.8-15.0) | | 83 | 83 | 1,409 | 5.9 (4.6-7.1) | |
| Hospitalization | 778 | 807 | 3,376 | 23.9 (22.3-25.3) | 474 | 503 | 2,868 | 17.5 (15.9-19.0) | 280 | 281 | 2,022 | 13.9 (12.3-15.4) | | 170 | 171 | 1,409 | 12.1 (10.2-14.0) | |
| **HFuEF** | | | | | | | | | | | | | | | | | | |
| **All-cause HCRU** | | | | | | | | | | | | | | | | | | |
| Outpatient Visits | 4,819 | 46,992 | 4,974 | 944.8 (934.5-955.0) | 4,702 | 34,702 | 4,262 | 814.3 (802.2-827.2) | 3,342 | 22,634 | 2,913 | 777.1 (750.8-803.8) | | 2,230 | 13,968 | 1,949 | 716.8 (681.6-752.4) | |
| GPs visits | 4,666 | 41,209 | 4,974 | 828.5 (818.5-837.6) | 4,624 | 31,298 | 4,262 | 734.4 (723.1-747.4) | 3,342 | 22,125 | 2,913 | 759.6 (733.6-786.4) | | 2,201 | 12,754 | 1,949 | 654.5 (621.9-687.4) | |
| Specialist visits | 2,273 | 5,783 | 4,974 | 116.3 (112.2-120.5) | 1,461 | 3,404 | 4,262 | 79.9 (76.0-83.5) | 843 | 1,988 | 2,913 | 68.3 (63.3- 73.6) | | 517 | 1,214 | 1,949 | 62.3 (56.7-68.5) | |
| Hospitalization | 1,332 | 1,475 | 4,974 | 29.7 (28.1-31.1) | 882 | 967 | 4,262 | 22.7 (21.2-24.1) | 477 | 486 | 2,913 | 16.7 (15.3-18.2) | | 292 | 295 | 1,949 | 15.1 (13.4-16.9) | |
| **HF-related HCRU** | | | | | | | | | | | | | | | | | | |
| Outpatient Visits | 4,797 | 34,914 | 4,974 | 701.9 (694.1-709.6) | 4661 | 24,638 | 4,262 | 578.1 (569.4-588.0) | 3342 | 16,268 | 2,913 | 558.5 (539.6- 577.8) | | 2,174 | 8,730 | 1,949 | 448.0 (425.3-470.2) | |
| GPs visits | 4,665 | 32,000 | 4,974 | 643.4 (635.4-650.9) | 4591 | 23,487 | 4,262 | 551.1 (542.0-561.0) | 3309 | 15,871 | 2,913 | 544.9 (526.0-563.7) | | 2,174 | 8,662 | 1,949 | 444.5 (421.4-466.2) | |
| Specialist visits | 2,009 | 2,914 | 4,974 | 58.6 (56.5-60.8) | 1034 | 1,151 | 4,262 | 27.0 (25.5-28.5) | 397 | 397 | 2,913 | 13.6 (12.3-14.9) | | 68 | 68 | 1,949 | 3.5 (2.6- 4.4) | |
| Hospitalization | 1,197 | 1,261 | 4,974 | 25.4 (24.1-26.7) | 772 | 815 | 4,262 | 19.1 (17.8-20.5) | 412 | 414 | 2,913 | 14.2 (13.0-15.5) | | 244 | 246 | 1,949 | 12.6 (11.0-14.2) | |

Inpatient and outpatient visits with a HF code as the primary code were assumed to be HF-related HCRU, including the first hospitalization for HF at index date

CI = Confidence interval, HF = Heart failure, HFmrEF = Heart failure with mildly reduced ejection fraction, HFpEF = Heart Failure with preserved ejection fraction, HFrEF = Heart failure with reduced ejection fraction, HFuEF = Heart Failure with unspecified ejection, HRCU = Healthcare resource utilization.

**Supplementary Table 3.** **Overall Healthcare Resource Costs per Patients by Years since Index in the Incident HF Cohort.**

|  | **Year 1 (N = 19,961)** | | | **Year 2 (N = 19,338)** | | | **Year 3 (N = 14,381)** | | | **Year 4 (N = 9,812)** | | |
| --- | --- | --- | --- | --- | --- | --- | --- | --- | --- | --- | --- | --- |
|  | **Number of Patients** | **Total cost, € (% on overall cost)** | **Mean Cost per patient (SD), €** | **Number of Patients** | **Total cost, € (% on overall cost)** | **Mean Cost per patient (SD), €** | **Number of Patients** | **Total cost, € (% on overall cost)** | **Mean Cost per patient (SD), €** | **Number of Patients** | **Total cost, € (% on overall cost)** | **Mean Cost per patient (SD), €** |
|  |  |  |  |  |  |  |  |  |  |  |  |  |
| **All HF patients** | | | | | | | | | | | | |
| **Outpatient** | 19961 | 8,114,347.4 (12.4%) | 406.5 (224.0) | 19,338 | 6,534,013.5  (14.2%) | 337.9 (226.6) | 14,381 | 3,868,111.6 (13.6%) | 269.0 (199.2) | 9,812 | 2,427,454.9 (12.8%) | 247.4 (176.6) |
| GPs visits^1^ | 19961 | 5,833,792.4 (8.9%) | 292.3 (164.8) | 19,338 | 4,981,513.5 (10.8%) | 257.6 (175.7) | 14,381 | 3,021,054.1 (10.6%) | 210.1 (154.6) | 9,812 | 1,925,187.4 (10.2%) | 196.2 (140.4) |
| Specialist visits^1^ | 19961 | 2,280,555.0 (3.5%) | 114.3 (115.1) | 19,338 | 1,552,500.0 (3.4%) | 80.3 (107.3) | 14,381 | 847,057.5 (3.0%) | 58.9 (87.5) | 9,812 | 502,267.5 (2.6%) | 51.2 (70.2) |
| **Inpatient** |  |  |  |  |  |  |  |  |  |  |  |  |
| Hospitalizations (>24 hours)^2^ | 19961 | 34,960,450.5 (53.5%) | 1,751.4 (2975.4) | 19,338 | 16,733,651.4 (36.4%) | 865.3 (2116.4) | 14,381 | 9,452,109.5 (33.1%) | 657.3 (1510.1) | 9,812 | 5,506,644.0 (29.1%) | 561.2 (1,147.9) |
| **Pharmacy** |  |  |  |  |  |  |  |  |  |  |  |  |
| Prescriptions^3^ | 19961 | 13,921,972.8 (21.3%) | 697.5 (240.5) | 19,338 | 11,912,331.3 (25.9%) | 616.0 (297.8) | 14,381 | 8,319,179.2 (29.1%) | 578.5 (363.6) | 9,812 | 5,635,689.2 (29.7%) | 574.4 (354.3) |
| **Indirect cost** |  |  |  |  |  |  |  |  |  |  |  |  |
| Cost of absence from work^4^ | 19961 | 8,368,852.5 (12.8%) | 4,19.3 (1360.1) | 19,338 | 10,774,614.2 (23.4%) | 557.2 (1,620.5) | 14,381 | 6,904,343.8 (24.2%) | 480.1 (,1126.4) | 9,812 | 5,378,906.7 (28.4%) | 548.2 (925.1) |
| **Total Overall cost** |  | 65,365,623.2 | 3,274.7 (4,053.4) |  | 45,954,610.3 | 2376.4 (3,434.3) |  | 28,543,744.0 | 1984.8 (2,472.8) |  | 18,948,694.8 | 1,931.2 (1,949.1) |
| **HFrEF** | | | | | | | | | | | | |
| **Outpatient** | 8678 | 5,147,626.1 (10.4%) | 593.2 (131.2) | 8,395 | 4,270,402.1 (14.4%) | 508.7 (118.2) | 6,250 | 2,447,683.1 (12.9%) | 391.6 (120.5) | 4,361 | 1,560,352.0 (12.3%) | 357.8 (106.5) |
| GPs visits^1^ | 8678 | 3,775,216.1 (7.6%) | 435.0 (83.8) | 8,395 | 3,276,329.6 (11.1%) | 390.3 (80.8) | 6,250 | 1,904,038.1 (10.0%) | 304.6 (89.2) | 4,361 | 1,246,949.5 (9.8%) | 285.9 (78.8) |
| Specialist visits^1^ | 8678 | 1,372,410.0 (2.8%) | 158.1 (99.6) | 8,395 | 994,072.5 (3.3%) | 118.4 (84.2) | 6,250 | 543,645.0 (2.9%) | 87.0 (66.3) | 4,361 | 313,402.5 (2.5%) | 71.9 (53.8) |
| **Inpatient** |  |  |  |  |  |  |  |  |  |  |  |  |
| Hospitalizations (>24 hours)^2^ | 8678 | 30,660,069.6 (62.1%) | 3,533.1 (1,102.5) | 8,395 | 11,317,501.2 (38.2%) | 1348.1 (1,280.2) | 6,250 | 6,727,668.5 (35.4%) | 1076.4 (868.2) | 4,361 | 3,875,188.4 (30.5%) | 888.6 (676.1) |
| **Pharmacy** |  |  |  |  |  |  |  |  |  |  |  |  |
| Prescriptions^3^ | 8678 | 6,579,254.4 (13.3%) | 758.2 (231.5) | 8,395 | 5,622,278.2 (19.0%) | 669.7 (281.2) | 6,250 | 3,978,415.2 (20.9%) | 636.5 (339.5) | 4,361 | 2,701,829.5 (21.3%) | 619.5 (332.2) |
| **Indirect cost** |  |  |  |  |  |  |  |  |  |  |  |  |
| Cost of absence from work^4^ | 8678 | 6,972,863.0 (14.1%) | 8,03.5 (694.1) | 8,395 | 8,427,756.7 (28.4%) | 1,003.9 (917.3) | 6,250 | 5,836,375.9 (30.7%) | 933.8 (451.8) | 4,361 | 4,564,267.4 (35.9%) | 1,046.6 (389.0) |
| **Total Overall cost** |  | 49,359,813.0 | 5,687.9 (1,581) |  | 29,637,938.2 | 3,530.4 (1,953.4) |  | 18,990,142.7 | 3,038.4 (1,249.8) |  | 12,701,637.3 | 2,912.6 (1,056.5) |
| **HFmrEF** | | | | | | | | | | | | |
| **Outpatient** | 1022 | 290,215.7 (16.3%) | 284.0 (226) | 991 | 215,761.1  (11.5%) | 217.7 (229.3) | 763 | 126,084.3  (12.0%) | 165.2 (202.0) | 474 | 74,815.2  (11.7%) | 157.8 (179.3) |
| GPs visits^1^ | 1022 | 198,483.2 (11.2%) | 194.2 (166.4) | 991 | 157,576.1 (8.4%) | 159.0 (178.0) | 763 | 95,844.3 (9.1%) | 125.6 (156.9) | 474 | 56,050.2  (8.7%) | 118.2 (142.8) |
| Specialist visits^1^ | 1022 | 91,732.5 (5.1%) | 89.8 (115.6) | 991 | 58,185.0 (3.1%) | 58.7 (108.1) | 763 | 30,240.0 (2.9%) | 39.6 (88.4) | 474 | 18,765.0  (2.9%) | 39.6 (70.9) |
| **Inpatient** |  |  |  |  |  |  |  |  |  |  |  |  |
| Hospitalizations (>24 hours)^2^ | 1022 | 678,061.7 (38.2%) | 663.5 (3,022.5) | 991 | 785,563.2 (41.8%) | 792.7 (2,128.0) | 763 | 406,580.3 (38.9%) | 532.9 (1,523.0) | 474 | 222,062.8  (34.7%) | 468.5 (1,157.6) |
| **Pharmacy** |  |  |  |  |  |  |  |  |  |  |  |  |
| Prescriptions^3^ | 1022 | 640,612.4 (36.1%) | 626.8 (241.2) | 991 | 568,773.3 (30.2%) | 573.9 (299.1) | 763 | 385,004.1 (36.8%) | 504.6 (365.4) | 474 | 243,526.7  (38.0%) | 513.8 (355.9) |
| **Indirect cost** |  |  |  |  |  |  |  |  |  |  |  |  |
| Cost of absence from work^4^ | 1022 | 167,603.8 (9.4%) | 164.0 (1,382.9) | 991 | 310,006.2 (16.5%) | 312.8 (1,639.5) | 763 | 128,941.5 (12.3%) | 169.0 (1,148.8) | 474 | 100,400.3  (15.7%) | 211.8 (940.3) |
| **Total Overall cost** |  | 1,776,493.6 | 1,738.3 (4,117.7) |  | 1,880,103.8 | 1,897.2 (3,462.2) |  | 1,046,610.2 | 1,371.7 (2,508.6) |  | 640,805.0 | 1,351.9 (1,971.6) |
| **HFpEF** | | | | | | | | | | | | |
| **Outpatient** | 5244 | 1,330,516.4 (18.9%) | 253.7 (225.4) | 5,074 | 1,092,279.7  (14.7%) | 215.3 (239.6) | 3,762 | 647,075.4  (14.7%) | 172.0 (215.1) | 2,598 | 414,577.4  (14.5%) | 159.6 (192.9) |
| GPs visits^1^ | 5244 | 904,456.4  (12.9%) | 172.5 (165.5) | 5,074 | 821,807.2  (11.1%) | 162.0 (187.0) | 3,762 | 508,092.9  (11.6%) | 135.1 (167.8) | 2,598 | 326,422.4  (11.4%) | 125.6 (154.2) |
| Specialist visits^1^ | 5244 | 426,060.0  (6.0%) | 81.2 (118.0) | 5,074 | 270,472.5  (3.6%) | 53.3 (1,12.5) | 3,762 | 138,982.5  (3.1%) | 36.9 (93.3) | 2,598 | 88,155.0  (3.1%) | 33.9 (74.9) |
| **Inpatient** |  |  |  |  |  |  |  |  |  |  |  |  |
| Hospitalizations (>24 hours)^2^ | 5244 | 1,858,973.7  (26.4%) | 354.5 (3,262.3) | 5,074 | 2,512,005.2  (33.8%) | 495.1 (2,326.4) | 3,762 | 1,333,981.3  (30.4%) | 354.6 (1,665) | 2,598 | 765,667.4  (26.8%) | 294.7 (1,268.2) |
| **Pharmacy** |  |  |  |  |  |  |  |  |  |  |  |  |
| Prescriptions^3^ | 5244 | 3,297,610.3  (46.9%) | 628.8 (242.2) | 5,074 | 2,806,253.7  (37.8%) | 553.1 (303.6) | 3,762 | 1,959,522.8  (44.7%) | 520.9 (374.3) | 2,598 | 1,351,995.0  (47.4%) | 520.4 (364.6) |
| **Indirect cost** |  |  |  |  |  |  |  |  |  |  |  |  |
| Cost of absence from work^4^ | 5244 | 545,623.1  (7.8%) | 104.0 (1,519.9) | 5,074 | 1,017,970.2  (13.7%) | 200.6 (1,793.5) | 3,762 | 446,437.3  (10.2%) | 118.7 (1,273.9) | 2,598 | 322,859.9  (11.3%) | 124.3 (1,046.6) |
| **Total Overall cost** |  | 7,032,723.5 | 1,341.1 (4,434.6) |  | 7,428,508.8 | 1,464 (3,778.6) |  | 4,387,016.8 | 1,166.1 (2,747.7) |  | 2,855,099.8 | 1,099 (2,164.8) |
| **HFpEF (50% to <60%)** | | | | | | | | | | | | |
| **Outpatient** | 1833 | 466,731.0  (18.3%) | 254.6 (225.7) | 1,774 | 386,375.1  (14.5%) | 217.8 (231.2) | 1,334 | 230,506.1  (15.2%) | 172.8 (204.2) | 902 | 146,535.4  (15.5%) | 162.5 (181.4) |
| GPs visits^1^ | 1833 | 316,543.5  (12.4%) | 172.7 (166.1) | 1,774 | 292,820.1  (11.0%) | 165.1 (179.8) | 1,334 | 179,003.6  (11.8%) | 134.2 (158.7) | 902 | 113,190.4  (12.0%) | 125.5 (144.6) |
| Specialist visits^1^ | 1833 | 150,187.5  (5.9%) | 81.9 (116) | 1,774 | 93,555.0  (3.5%) | 52.7 (108.8) | 1,334 | 51,502.5  (3.4%) | 38.6 (89.1) | 902 | 33,345.0  (3.5%) | 37.0 (71.4) |
| **Inpatient** |  |  |  |  |  |  |  |  |  |  |  |  |
| Hospitalizations (>24 hours)^2^ | 1833 | 721,062.3  (28.3%) | 393.4 (3,067.6) | 1,774 | 883,758.6  (33.2%) | 498.2 (2,179.6) | 1,334 | 462,737.8  (30.6%) | 346.9 (1,555.4) | 902 | 239,391.4  (25.3%) | 265.4 (1,184.9) |
| **Pharmacy** |  |  |  |  |  |  |  |  |  |  |  |  |
| Prescriptions^3^ | 1833 | 1,138,557.1  (44.7%) | 621.1 (241.1) | 1,774 | 971,907.0  (36.5%) | 547.9 (299.9) | 1,334 | 671,362.0  (44.4%) | 503.3 (367.6) | 902 | 454,684.5  (48.1%) | 504.1 (358.0) |
| **Indirect cost** |  |  |  |  |  |  |  |  |  |  |  |  |
| Cost of absence from work^4^ | 1833 | 218,310.0 (8.6%) | 1,19.1 (1,404.5) | 1,774 | 418,807.0  (15.7%) | 236.1 (1,665.5) | 1,334 | 149,082.3  (9.8%) | 111.8 (1,170.6) | 902 | 104,145.1  (11.0%) | 115.5 (961.1) |
| **Total Overall cost** |  | 2,544,660.4 | 1,388.2 (4,174.8) |  | 2,660,847.7 | 1,499.9 (3,534.9) |  | 1,513,688.2 | 1,134.7 (2,554.8) |  | 944,756.4 | 1,047.4 (2,013.3) |
| **HFpEF (≥60%)** | | | | | | | | | | | | |
| **Outpatient** | 3411 | 863,785.4  (19.2%) | 253.2 (226.4) | 3,300 | 705,904.6  (14.8%) | 213.9 (235.1) | 2,428 | 416,569.3  (14.5%) | 171.6 (208.9) | 1,696 | 268,042.1  (14.0%) | 158 (186.5) |
| GPs visits^1^ | 3411 | 587,912.9  (13.1%) | 172.4 (166.6) | 3,300 | 528,987.1  (11.1%) | 160.3 (182.8) | 2,428 | 329,089.3  (11.5%) | 135.5 (162.6) | 1,696 | 213,232.1  (11.2%) | 125.7 (148.7) |
| Specialist visits^1^ | 3411 | 275,872.5  (6.1%) | 80.9 (117) | 3,300 | 176,917.5  (3.7%) | 53.6 (110.5) | 2,428 | 87,480.0  (3.0%) | 36.0 (91.0) | 1,696 | 54,810.0  (2.8%) | 32.3 (73.1) |
| **Inpatient** |  |  |  |  |  |  |  |  |  |  |  |  |
| Hospitalizations (>24 hours)^2^ | 3411 | 1,137,911.4  (25.4%) | 333.6 (3,154.0) | 3,300 | 1,628,246.6  (34.2%) | 493.4 (2,240.5) | 2,428 | 871,243.5  (30.3%) | 358.8 (1,602.7) | 1,696 | 526,276.0  (27.5%) | 310.3 (1,217.7) |
| **Pharmacy** |  |  |  |  |  |  |  |  |  |  |  |  |
| Prescriptions^3^ | 3411 | 2,159,053.2  (48.1%) | 633.0 (242.0) | 3,300 | 1,834,346.7  (38.5%) | 555.9 (301.0) | 2,428 | 1,288,160.9  (44.8%) | 530.5 (369.0) | 1,696 | 897,310.5  (47.0%) | 529.1 (359.5) |
| **Indirect cost** |  |  |  |  |  |  |  |  |  |  |  |  |
| Cost of absence from work^4^ | 3411 | 327,313.1  (7.3%) | 96.0 (1,459.6) | 3,300 | 599,163.2  (12.5%) | 181.6 (1,731.0) | 2,428 | 297,355.0  (10.3%) | 122.5 (1,215.2) | 1,696 | 218,714.8  (11.4%) | 129.0 (998.5) |
| **Total Overall cost** |  | 4,488,063.1 | 1,315.8 (4,293.1) |  | 4,767,661.10 | 1,444.7 (3,644.3) |  | 2,873,328.6 | 1.183.4 (2,638.3) |  | 1,910,343.3 | 1,126.4 (2,078.3) |
| **HFuEF** | | | | | | | | | | | | |
| **Outpatient** | 5017 | 1,345,989.2  (18.7%) | 268.3 (230.5) | 4,878 | 955,570.6  (13.6%) | 195.9 (237.1) | 3,606 | 647,268.8  (15.7%) | 179.5 (214.9) | 2,379 | 377,710.3  (13.7%) | 158.8 (192.3) |
| GPs visits^1^ | 5017 | 955,636.7  (13.3%) | 190.5 (172.0) | 4,878 | 725,800.6  (10.4%) | 148.8 (186.1) | 3,606 | 513,078.8  (12.5%) | 142.3 (167.7) | 2,379 | 295,765.3  (10.8%) | 124.3 (154.1) |
| Specialist visits^1^ | 5017 | 390,352.5  (5.4%) | 77.8 (117.4) | 4,878 | 229,770.0  (3.2%) | 47.1 (112.7) | 3,606 | 134,190.0  (3.2%) | 37.2 (92.9) | 2,379 | 81,945.0  (2.9%) | 34.4 (74.8) |
| **Inpatient** |  |  |  |  |  |  |  |  |  |  |  |  |
| Hospitalizations (>24 hours)^2^ | 5017 | 1,763,345.5  (24.5%) | 351.5 (3,257.1) | 4,878 | 2,118,581.8  (30.2%) | 434.3 (2,345.6) | 3,606 | 983,879.4  (23.9%) | 272.8 (1,684.9) | 2,379 | 643,725.4  (23.4%) | 270.6 (1,277.7) |
| **Pharmacy** |  |  |  |  |  |  |  |  |  |  |  |  |
| Prescriptions^3^ | 5017 | 3,404,495.7  (47.3%) | 678.6 (242.6) | 4,878 | 2,915,026.0  (41.6%) | 597.6 (301.0) | 3,606 | 1,996,237.1  (48.4%) | 553.6 (367.1) | 2,379 | 1,338,338.0  (48.6%) | 562.6 (357.3) |
| **Indirect cost** |  |  |  |  |  |  |  |  |  |  |  |  |
| Cost of absence from work^4^ | 5017 | 682,762.7  (9.5%) | 136.1 (1,503.0) | 4,878 | 1,018,881.1  (14.5%) | 208.9 (1,788.2) | 3,606 | 492,589.1  (12.0%) | 136.6 (1,262.6) | 2,379 | 391,379.1  (14.2%) | 164.5 (1,036.9) |
| **Total Overall cost** |  | 7,196,593.10 | 1,434.4 (4,432.2) |  | 7,008,059.5 | 1,436.7 (3,791.2) |  | 4,119,974.3 | 1,142.5 (2,749.9) |  | 2,751,152.8 | 1,156.4 (2,162.2) |

1. Costs were calculated based on standard cost for GP/ specialist visits; 2. Costs of inpatient stays were calculated based on the Diagnosis Related Group (DRG)-based reimbursement of the stays; 3. Costs based on full price of product; 4. Costs of absence from work were calculated by multiplying the number of days of absence from work due to sickness by the mean daily salary of a working person in Germany, obtained from the Federal Institute for Occupational Safety and Health. All cost were presented in euros

GP = General practitioner, HF = Heart failure, HFmrEF = Heart failure with mildly reduced ejection fraction, HFpEF = Heart Failure with preserved ejection fraction, HFrEF = Heart failure with reduced ejection fraction, HFuEF = Heart Failure with unspecified ejection, HRCU = Healthcare resource utilization, IQR = Interquartile range, SD = Standard deviation.

**Supplementary Table 4. HF-related Healthcare Resource Costs per Patients by Years since Index in the Incident HF Cohort**

|  | **Year 1 (N = 19,961)** | | | **Year 2 (N = 19,338)** | | | **Year 3 (N = 14,381)** | | | **Year 4 (N = 9,812)** | | |
| --- | --- | --- | --- | --- | --- | --- | --- | --- | --- | --- | --- | --- |
|  | **All patients** | | | **All patients** | | | **All patients** | | | **All patients** | | |
|  | **Number of Patients** | **Total cost, € (% on overall cost)** | **Mean Cost per patient (SD), €** | **Number of Patients** | **Total cost, € (% on overall cost)** | **Mean Cost per patient (SD), €** | **Number of Patients** | **Total cost, € (% on overall cost)** | **Mean Cost per patient (SD), €** | **Number of Patients** | **Total cost, € (% on overall cost)** | **Mean Cost per patient (SD), €** |
|  |  |  |  |  |  |  |  |  |  |  |  |  |
| **All HF patients** | | | | | | | | | | | | |
| **Outpatient** | 19,961 | 6,328,383.7  (12.6%) | 317.0 (194.1) | 19,338 | 4,798,816.6  (15.3%) | 248.2 (180.6) | 14,381 | 2,656,931.5  (14.1%) | 184.8 (143.7) | 9,812 | 1,560,210.0  (12.9%) | 159.0 (119.1) |
| GPs visits^1^ | 19,961 | 4,712,231.2  (9.4%) | 236.1 (140.4) | 19,338 | 3,844,299.1  (12.3%) | 198.8 (140.4) | 14,381 | 2,216,291.5  (11.8%) | 154.1 (115.8) | 9,812 | 1,348,710.0  (11.2%) | 137.5 (100.4) |
| Specialist visits^1^ | 19,961 | 1,616,152.5  (3.2%) | 81.0 (90.5) | 19,338 | 954,517.5  (3.0%) | 49.4 (75.6) | 14,381 | 440,640.0  (2.3%) | 30.6 (53.9) | 9,812 | 211,410.0  (1.7%) | 21.5 (37.8) |
| **Inpatient** |  |  |  |  |  |  |  |  |  |  |  |  |
| Hospitalizations (>24 hours)^2^ | 19,961 | 31,686,628.7  (63.3%) | 1,587.4 (2,669.8) | 19,338 | 13,938,933.3  (44.5%) | 720.8 (1,857.1) | 14,381 | 7,962,812.6  (42.3%) | 553.7 (1,321.8) | 9,812 | 4,632,512.4  (38.2%) | 472.1 (1,010.1) |
| **Pharmacy** |  |  |  |  |  |  |  |  |  |  |  |  |
| Prescriptions^3^ | 19,961 | 5,947,807.5  (11.9%) | 298.0 (140.7) | 19,338 | 5,086,750.8  (16.2%) | 263.0 (153.8) | 14,381 | 3,547,173.4  (18.8%) | 246.7 (1,70.5) | 9,812 | 2,410,166.6  (19.9%) | 245.6 (162.3) |
| **Indirect cost** |  |  |  |  |  |  |  |  |  |  |  |  |
| Cost of absence from work^4^ | 19,961 | 6,131,708.0  (12.2%) | 307.2 (1,089.1) | 19,338 | 7,481,412.4  (23.9%) | 386.9 (1,238.9) | 14,381 | 4,651,556.8  (24.7%) | 323.5 (856.6) | 9,812 | 3,510,729.2  (29.0%) | 357.8 (687.2) |
| **Total Overall cost** |  | 50,094,527.9 | 2,509.6 (3,518.5) |  | 31,305,913.0 | 1,618.9 (2,840.2) |  | 18,818,474.3 | 1,308.6 (1,994.4) |  | 12,113,618.2 | 1,234.6 (1,534.1) |
| **HFrEF** | | | | | | | | | | | | |
| **Outpatient** | 8,678 | 4,250,185.4  (10.6%) | 489.8 (87.7) | 8,395 | 3,314,898.5  (14.7%) | 394.9 (72.7) | 6,250 | 1,785,828.9  (12.9%) | 285.7 (70.7) | 4,361 | 1,081,169.5  (12.0%) | 247.9 (56.7) |
| GPs visits^1^ | 8,678 | 3,098,230.4  (7.7%) | 357.0 (68.5) | 8,395 | 2,555,051.0  (11.4%) | 304.4 (63.4) | 6,250 | 1,408,166.4  (10.2%) | 225.3 (65.9) | 4,361 | 885,487.0  (9.8%) | 203.0 (54.7) |
| Specialist visits^1^ | 8,678 | 1,151,955.0  (2.9%) | 132.7 (53.7) | 8,395 | 759,847.5  (3.3%) | 90.5 (34.0) | 6,250 | 377,662.5  (2.7%) | 60.4 (18.6) | 4,361 | 195,682.5  (2.2%) | 44.9 (9.6) |
| **Inpatient** |  |  |  |  |  |  |  |  |  |  |  |  |
| Hospitalizations (>24 hours)^2^ | 8,678 | 27,505,943.5  (68.6%) | 3,169.6 (941.1) | 8,395 | 10,174,134.5  (45.2%) | 1,211.9 (922.7) | 6,250 | 6,020,725.8  (43.5%) | 963.3 (635.2) | 4,361 | 3,479,197.8  (38.5%) | 797.8 (499.3) |
| **Pharmacy** |  |  |  |  |  |  |  |  |  |  |  |  |
| Prescriptions^3^ | 8,678 | 2,777,983.6  (6.9%) | 320.1 (138.1) | 8,395 | 2,370,542.4  (10.5%) | 282.4 (148.8) | 6,250 | 1,677,156.2  (12.1%) | 268.3 (162.2) | 4,361 | 1,142,748.0  (12.6%) | 262.0 (154.8) |
| **Indirect cost** |  |  |  |  |  |  |  |  |  |  |  |  |
| Cost of absence from work^4^ | 8,678 | 5,558,308.8  (13.9%) | 640.5 (330.1) | 8,395 | 6,636,493.6  (29.5%) | 790.5 (391.0) | 6,250 | 4,350,790.4  (31.4%) | 696.1 (154.5) | 4,361 | 3,334,944.8  (36.9%) | 764.7 (106.0) |
| **Total Overall cost** |  | 40,092,421.3 | 4,620 (1,087.4) |  | 22,496,069.0 | 2,679.7 (1,175.3) |  | 13,834,501.1 | 2,213.5 (756.6) |  | 9,038,060.1 | 2,072.5 (610.3) |
| **HFmrEF** | | | | | | | | | | | | |
| **Outpatient** | 1,022 | 210,097.3  (19.7%) | 205.6 (196.3) | 991 | 143,320.2  (12.9%) | 144.6 (183.0) | 763 | 77,951.6  (13.3%) | 102.2 (146.0) | 474 | 41,855.1  (12.6%) | 88.3 (121.3) |
| GPs visits^1^ | 1,022 | 157,784.8  (14.8%) | 154.4 (141.9) | 991 | 121,585.2  (11.0%) | 122.7 (142.4) | 763 | 71,471.6  (12.2%) | 93.7 (117.6) | 474 | 39,492.6  (11.8%) | 83.3 (102.1) |
| Specialist visits^1^ | 1,022 | 52,312.5  (4.9%) | 51.2 (91.5) | 991 | 21,735.0  (1.9%) | 21.9 (76.8) | 763 | 6,480.0  (1.1%) | 8.5 (55.1) | 474 | 2,362.5  (0.9%) | 5.0 (38.6) |
| **Inpatient** |  |  |  |  |  |  |  |  |  |  |  |  |
| Hospitalizations (>24 hours)^2^ | 1,022 | 491,939.7  (46.0%) | 481.4 (2,715.6) | 991 | 567,672.1  (51.3%) | 572.8 (1,879.6) | 763 | 297,474.3  (50.9%) | 389.9 (1,339.9) | 474 | 159,487.3  (47.8%) | 336.5 (1024.7) |
| **Pharmacy** |  |  |  |  |  |  |  |  |  |  |  |  |
| Prescriptions^3^ | 1,022 | 278,289.9  (26.1%) | 272.3 (141.1) | 991 | 246,993.4  (22.3%) | 249.2 (154.2) | 763 | 165,273.4  (28.3%) | 216.6 (171.2) | 474 | 104,690.8  (31.4%) | 220.9 (162.9) |
| **Indirect cost** |  |  |  |  |  |  |  |  |  |  |  |  |
| Cost of absence from work^4^ | 1,022 | 87,639.2  (8.2%) | 85.8 (1,111.4) | 991 | 149,573.6  (13.5%) | 150.9 (1,261.8) | 763 | 44,123.2  (7.5%) | 57.8 (876.6) | 474 | 27,324.0  (8.2%) | 57.6 (703.2) |
| **Total Overall cost** |  | 1,067,966.1 | 1,045 (3,580.3) |  | 1,107,559.2 | 1,117.6 (2,881.3) |  | 584,822.4 | 766.5 (2,030.6) |  | 333,357.2 | 703.3 (1,561.1) |
| **HFpEF** | | | | | | | | | | | | |
| **Outpatient** | 5,244 | 929,326.1  (25.6%) | 177.2 (197.5) | 5,074 | 718,241.9  (19.2%) | 141.6 (192.2) | 3,762 | 398,305.1  (18.8%) | 105.9 (157) | 2,598 | 231,723.7  (17.5%) | 89.2 (132.1) |
| GPs visits^1^ | 5,244 | 714,136.1  (19.7%) | 136.2 (142.3) | 5,074 | 622,999.5  (16.7%) | 122.8 (149.8) | 3,762 | 368,605.1  (17.4%) | 98 (125.8) | 2,598 | 222,948.7  (16.8%) | 85.8 (110.5) |
| Specialist visits^1^ | 5,244 | 215,190.0  (5.9%) | 41.0 (96.7) | 5,074 | 95,242.5  (2.5%) | 18.8 (83) | 3,762 | 29,700.0  (1.4%) | 7.9 (60.7) | 2,598 | 8,775.0  (0.7%) | 3.4 (43.1) |
| **Inpatient** |  |  |  |  |  |  |  |  |  |  |  |  |
| Hospitalizations (>24 hours)^2^ | 5,244 | 1,090,739.1  (30.0%) | 208.0 (2,931.2) | 5,074 | 1,478,707.2  (39.5%) | 291.4 (2,092.9) | 3,762 | 782,033.3  (36.8%) | 207.9 (1,495.1) | 2,598 | 456,640.7  (34.5%) | 175.8 (1,145.6) |
| **Pharmacy** |  |  |  |  |  |  |  |  |  |  |  |  |
| Prescriptions^3^ | 5,244 | 1,386,225.9  (38.2%) | 264.3 (141.4) | 5,074 | 1,181,633.9  (31.6%) | 232.9 (156.2) | 3,762 | 823,380.5  (38.8%) | 218.9 (175.3) | 2,598 | 570,940.4  (43.1%) | 219.8 (166.7) |
| **Indirect cost** |  |  |  |  |  |  |  |  |  |  |  |  |
| Cost of absence from work^4^ | 5,244 | 225,574.8  (6.2%) | 43.0 (1,241.2) | 5,074 | 361,182.8  (9.7%) | 71.2 (1,409.8) | 3,762 | 118,910.0  (5.6%) | 31.6 (982.9) | 2,598 | 65,071.6  (4.9%) | 25.0 (790.2) |
| **Total Overall cost** |  | 3,631,865.8 | 692.6 (3,865.3) |  | 3,739,765.8 | 737 (3,196.8) |  | 2,122,628.8 | 564.2 (2,257.9) |  | 1,324,376.4 | 509.8 (1,741.8) |
| **HFpEF (50% to <60%)** | | | | | | | | | | | | |
| **Outpatient** | 1,833 | 326,202.3  (24.5%) | 178.0 (196.4) | 1,774 | 254,821.9  (19.1%) | 143.6 (184.8) | 1,334 | 140,881.4  (19.2%) | 105.6 (147.9) | 902 | 80,951.8  (18.3%) | 89.7 (123.1) |
| GPs visits^1^ | 1,833 | 249,454.8  (18.7%) | 136.1 (141.8) | 1,774 | 221,881.9  (16.6%) | 125.1 (143.8) | 1,334 | 129,608.9  (17.6%) | 97.2 (118.9) | 902 | 77,779.3  (17.6%) | 86.2 (103.4) |
| Specialist visits^1^ | 1,833 | 76,747.5  (5.8%) | 41.9 (92.5) | 1,774 | 32,940.0  (2.5%) | 18.6 (77.9) | 1,334 | 11,272.5  (1.6%) | 8.5 (56.0) | 902 | 3,172.5  (0.7%) | 3.5 (39.4) |
| **Inpatient** |  |  |  |  |  |  |  |  |  |  |  |  |
| Hospitalizations (>24 hours)^2^ | 1,833 | 430,006.0  (32.3%) | 234.6 (2,755.8) | 1,774 | 519,216.2  (38.8%) | 292.7 (1,928.4) | 1,334 | 272,123.2  (37.0%) | 204.0 (1,373.6) | 902 | 144,405.0  (32.7%) | 160.1 (1,051) |
| **Pharmacy** |  |  |  |  |  |  |  |  |  |  |  |  |
| Prescriptions^3^ | 1,833 | 481,282.1  (36.2%) | 262.6 (141.0) | 1,774 | 412,652.3  (30.9%) | 232.6 (154.6) | 1,334 | 284,281.6  (38.7%) | 213.1 (172.2) | 902 | 194,621.8  (44.0%) | 215.8 (163.7) |
| **Indirect cost** |  |  |  |  |  |  |  |  |  |  |  |  |
| Cost of absence from work^4^ | 1,833 | 93,407.6 (7.0%) | 51.0 (1,134.1) | 1,774 | 150,079.6 (11.2%) | 84.6 (1,289.3) | 1,334 | 37,444.0 (5.1%) | 28.1 (894.8) | 902 | 21,859.2  (4.9%) | 24.2 (718.3) |
| **Total Overall cost** |  | 1,330,898.0 | 726.1 (3,632.1) |  | 1,336,770.0 | 753.5 (2,948.5) |  | 734,730.2 | 550.8 (2,074.2) |  | 441,837.8 | 489.8 (1,596.5) |
| **HFpEF (≥60%)** | | | | | | | | | | | | |
| **Outpatient** | 3,411 | 603,123.7  (26.2%) | 176.8 (197.6) | 3,300 | 463,419.9  (19.3%) | 140.4 (188.1) | 2,428 | 257,423.6  (18.5%) | 106.0 (151.8) | 1,696 | 150,771.9  (17.1%) | 88.9 (127.0) |
| GPs visits^1^ | 3,411 | 464,681.2  (20.2%) | 136.2 (142.6) | 3,300 | 401,117.4  (16.7%) | 121.6 (146.3) | 2,428 | 238,996.1  (17.2%) | 98.4 (121.9) | 1,696 | 145,169.4  (16.4%) | 85.6 (106.4) |
| Specialist visits^1^ | 3,411 | 138,442.5  (6.0%) | 40.6 (94.4) | 3,300 | 62,302.5  (2.6%) | 18.9 (80.2) | 2,428 | 18,427.5  (1.3%) | 7.6 (58.1) | 1,696 | 5,602.5  (0.7%) | 3.3 (41.0) |
| **Inpatient** |  |  |  |  |  |  |  |  |  |  |  |  |
| Hospitalizations (>24 hours)^2^ | 3,411 | 660,733.1  (28.7%) | 193.7 (2,833.7) | 3,300 | 959,491.0  (39.9%) | 290.8 (1,998.0) | 2,428 | 509,910.1  (36.7%) | 210.0 (1,425.6) | 1,696 | 312,235.7  (35.3%) | 184.1 (1,090.4) |
| **Pharmacy** |  |  |  |  |  |  |  |  |  |  |  |  |
| Prescriptions^3^ | 3,411 | 904,943.8  (39.3%) | 265.3 (1,41.2) | 3,300 | 768,981.7  (32.0%) | 233.0 (155.2) | 2,428 | 539,098.8  (38.8%) | 222.0 (173.0) | 1,696 | 376,318.6  (42.6%) | 221.9 (164.7) |
| **Indirect cost** |  |  |  |  |  |  |  |  |  |  |  |  |
| Cost of absence from work^4^ | 3,411 | 132,167.2  (5.7%) | 38.7 (1,181.4) | 3,300 | 211,103.2  (8.8%) | 64.0 (1,342.9) | 2,428 | 81,466.0  (5.9%) | 33.6 (932.6) | 1,696 | 43,212.4  (4.9%) | 25.5 (749.1) |
| **Total Overall cost** |  | 2,300,967.8 | 674.6 (3,736.3) |  | 2,402,995.8 | 728.2 (3,055.8) |  | 1,387,898.6 | 571.6 (2,153.0) |  | 882,538.6 | 520.4 (1,659) |
| **HFuEF** | | | | | | | | | | | | |
| **Outpatient** | 5,017 | 938,775.0  (17.7%) | 187.1 (201.3) | 4,878 | 622,356.0  (15.7%) | 127.6 (190.1) | 3,606 | 394,846.0  (17.3%) | 109.5 (156.8) | 2,379 | 205,461.8  (14.5%) | 86.4 (131.7) |
| GPs visits^1^ | 5,017 | 742,080.0  (14.0%) | 147.9 (146.5) | 4,878 | 544,663.5  (13.7%) | 111.7 (148.9) | 3,606 | 368,048.5  (16.2%) | 102.1 (125.8) | 2,379 | 200,871.8  (14.2%) | 84.4 (110.3) |
| Specialist visits^1^ | 5,017 | 196,695.0  (3.7%) | 39.2 (96.1) | 4,878 | 77,692.5  (2.0%) | 15.9 (82.6) | 3,606 | 26,797.5  (1.1%) | 7.4 (60.4) | 2,379 | 4,590.0  (0.3%) | 1.9 (42.9) |
| **Inpatient** |  |  |  |  |  |  |  |  |  |  |  |  |
| Hospitalizations (>24 hours)^2^ | 5,017 | 2,598,006.4  (49.0%) | 517.8 (2,931.7) | 4,878 | 1,718,419.5  (43.4%) | 352.3 (2,060) | 3,606 | 862,579.2  (37.9%) | 239.2 (1,471.1) | 2,379 | 537,186.6  (37.9%) | 225.8 (1,121.5) |
| **Pharmacy** |  |  |  |  |  |  |  |  |  |  |  |  |
| Prescriptions^3^ | 5,017 | 1,505,308.1  (28.4%) | 300.0 (140.9) | 4,878 | 1,287,581.1  (32.5%) | 264.0 (154.1) | 3,606 | 881,363.5  (38.7%) | 244.4 (170.4) | 2,379 | 591,787.4  (41.7%) | 248.8 (162.0) |
| **Indirect cost** |  |  |  |  |  |  |  |  |  |  |  |  |
| Cost of absence from work^4^ | 5,017 | 260,185.2  (4.9%463) | 51.9 (1,232.9) | 4,878 | 334,162.4  (8.4%) | 68.5 (1,402.6) | 3,606 | 137,733.2  (6.1%) | 38.2 (976.3) | 2,379 | 83,388.8  (5.9%) | 35.1 (784.7) |
| **Total Overall cost** |  | 5,302,274.7 | 1,056.9 (3,887.3) |  | 3,962,519.0 | 812.3 (3,171.4) |  | 2,276,521.9 | 631.3 (2,236.3) |  | 1,417,824.6 | 596.0 (1,719.5) |

1. Costs were calculated based on standard cost for GP/ specialist visits; 2. Costs of inpatient stays were calculated based on the Diagnosis Related Group (DRG)-based reimbursement of the stays; 3. Costs based on full price of product; 4. Costs of absence from work were calculated by multiplying the number of days of absence from work due to sickness by the mean daily salary of a working person in Germany, obtained from the Federal Institute for Occupational Safety and Health. All cost were presented in euros

GP = General practitioner, HF = Heart failure, HFmrEF = Heart failure with mildly reduced ejection fraction, HFpEF = Heart Failure with preserved ejection fraction, HFrEF = Heart failure with reduced ejection fraction, HFuEF = Heart Failure with unspecified ejection, HRCU = Healthcare resource utilization, IQR = Interquartile range, SD = Standard deviation.
